# Identification of Key Aroma Substances in Pomegranate from Different Geographical Origins via Integrated Volatile Profiling and Multivariate Statistical Analysis

**DOI:** 10.3390/foods14203546

**Published:** 2025-10-17

**Authors:** Yanzhen Zhang, Wenzhu Guo, Haitao Qu, Lihua Zhang, Lingxiao Liu, Xiaojie Hu, Yunguo Liu

**Affiliations:** 1 College of Life Sciences, Linyi University, Linyi 276005, China; 2 Department of Biotechnology, College of Engineering, The University of Suwon, Hwaseong 18323, Republic of Korea; 3 College of Food Science and Pharmaceutical Engineering, Zaozhuang University, Zaozhuang 277160, China; 4 Linyi Academy of Agricultural Sciences, Linyi 276000, China

**Keywords:** pomegranate, volatile compounds, HS-GC-IMS, HS-SPME-GC-MS, OPLS-DA

## Abstract

Pomegranate (*Punica granatum* L.), valued for its health benefits and distinctive flavor, derives its characteristic aroma from volatile organic compounds (VOCs) that vary significantly with geographical origin. In this study, VOCs in pomegranates from six Chinese geographical regions were characterized using an electronic nose (E-nose), an electronic tongue (E-tongue), headspace gas chromatography–ion mobility spectrometry (HS-GC-IMS), and headspace solid-phase microextraction–gas chromatography–mass spectrometry (HS-SPME-GC-MS). To elucidate geographical variations in odor, taste, and volatile profiles, a comprehensive multivariate statistical analysis integrating principal component analysis (PCA), hierarchical cluster analysis, orthogonal partial least squares-discriminant analysis (OPLS-DA), and variable importance in projection (VIP) was employed. The results demonstrated that the E-nose and E-tongue effectively distinguished pomegranate by geographical origin, with aroma contributing more significantly than taste to regional differentiation. A total of 46 and 58 VOCs were identified using HS-GC-IMS and HS-SPME-GC-MS, respectively, with different characteristic volatile compounds in pomegranate from various origins, and alkenes, esters, and alcohols were the primary contributors to regional variations. Notably, OPLS-DA revealed that HS-GC-IMS exhibited superior discriminatory power in separating pomegranates of different geographical origins, with HY and HL displaying closely related odor profiles while the other samples showed the most pronounced odor differences, but these findings contrasted with HS-SPME-GC-MS results. Additionally, the VIP method and the relative odor activity value (ROAV) further identified six and eight key aroma compounds based on HS-GC-IMS and HS-SPME-GC-MS data; in particular, hexanal, nonanal, *β*-pinene, 3-hydroxybutan-2-one, and *β*-ocimene were identified as key aroma compounds in pomegranate as potential regional markers. These findings highlight VOC profiles as potential geographical origin markers, supporting origin traceability and quality control in the pomegranate industry.

## 1. Introduction

As a fruit-bearing deciduous shrub, the pomegranate (*Punica granatum* L.) belongs to the *Punica* genus within the *Punicaceae* family and is native to regions extending from the Balkan Peninsula to Iran and its surrounding areas [1]. With over two millennia of cultivation history and diverse climatic conditions, China has become one of the world’s leading pomegranate producers, with well-known production areas including the provinces of Yunnan, Anhui, Shaanxi, Xinjiang, Sichuan, and Shandong [2]. Pomegranates are economically and medicinally significant fruits, appreciated for their unique flavor and potential health benefits. They have been utilized for thousands of years in traditional Chinese medicine and are listed in China’s catalog of “medicine and food homology” [3]. The edible portion of the pomegranate is commonly consumed fresh or processed into various industrial food products, including juice, beverages, jams, jellies, and flavoring or coloring drinks [4], while also being widely utilized in therapeutic formulations, food seasonings, and even cosmetics [5]. In addition, historically regarded as a “healing food,” pomegranates have demonstrated multiple beneficial effects on various diseases [6]. Recent scientific studies have proven that pomegranates’ bioactive ingredients and pharmacological effects can help treat cardiovascular diseases, diabetes, hyperlipidemia, hypertension, cancer, and more [7,8].

Volatile aroma is indisputably a critical sensory attribute in assessing pomegranate quality and shaping consumer perception. Nevertheless, fresh pomegranate fruit typically exhibits low aromatic intensity, making the extraction and identification of its volatile compounds challenging [9]. Furthermore, the volatile composition of pomegranates is significantly influenced by geographical origin, as climate, soil conditions, and cultivation practices vary across different regions, ultimately affecting their aromatic characteristics [10,11]. Understanding these variations is essential for both producers and consumers, as aroma profiles contribute to quality differentiation and market value. Despite the importance of volatile compounds in assessing pomegranate quality, existing domestic and international research has primarily focused on processed pomegranate products, while studies on the volatile compositions of fresh pomegranates remain limited [12]. In particular, investigations into aroma-active compounds across different production regions are scarce. Consequently, comprehensive profiling of VOCs in fresh pomegranates from diverse geographical origins is essential to elucidate the relationship between regional factors and aroma profiles and provides valuable insights into origin authentication.

In recent years, advanced sensory technologies such as the electronic nose (E-nose) and electronic tongue (E-tongue) have enabled rapid and objective evaluation of food aroma and taste attributes [13]. However, these systems cannot provide the specific volatile compounds responsible for organoleptic quality. In contrast, headspace-gas chromatography-ion mobility spectrometry (HS-GC-IMS) and headspace solid-phase microextraction gas chromatography–mass spectrometry (HS-SPME-GC-MS) can address this limitation by enabling the identification of key aroma-active compounds [14,15]. HS-GC-IMS has been widely applied for the comprehensive analysis of volatile aroma compounds in foods, with high sensitivity and low detection limit [16,17]. Similarly, HS-SPME-GC-MS combines the separation power with the identification capability of mass spectrometry, providing high sensitivity and strong qualitative and quantitative analytical performance [18]. The combined use of HS-GC-IMS and HS-SPME-GC-MS effectively expands the detection range of volatile components and enhances the accuracy and reliability of flavor analysis [19].

Therefore, in this study, the overall aroma profile and taste of pomegranate from six major growing regions were characterized using an E-nose and E-tongue. Meanwhile, the volatile compounds were analyzed using HS-GC-IMS and HS-SPME-GC-MS, and the key aroma compounds in pomegranate were identified based on relative odor activity values (ROAVs) and multivariate statistical analysis. These findings enhance the understanding of volatile compound variability in pomegranates across different regions, thereby supporting origin traceability and quality assurance within the pomegranate industry.

## 2. Materials and Methods

### 2.1. Experimental Materials

The pomegranate samples were sourced from six different growing areas in China, including MZ (Mengzi, Yunnan), HY (Huaiyuan, Anhui), LT (Lintong, Shanxi), TNS (Hetian, Xinjiang), HL (Huili, Sichuan), and HZZ (Zaozhuang, Shandong). All of the pomegranate samples are designated as National Geographic Indication Products, and detailed information about the samples is provided in Table 1. For each cultivar (with no obvious cracks or damage on the surface), we carefully removed the pomegranate seeds from the fruit, ensuring that the samples were suitable for subsequent analysis. For each experiment, 5 g of pomegranate puree samples were weighed, and each sample was analyzed in triplicate.

### 2.2. E-Nose Analysis

The analysis of pomegranate samples was performed using the cNose E-nose system (Shanghai Baosheng Industrial Development Co., Ltd., Shanghai, China), which is equipped with 14 metal oxide sensors designed to detect a wide range of volatile compounds. These sensors include S1 (propane, smoke), S2 (alcohol, smoke, isobutane, formaldehyde), S3 (ozone), S4 (hydrogen sulfide), S5 (ammonia), S6 (toluene, acetone, ethanol, hydrogen), S7 (methane, natural gas, biogas), S8 (liquefied gas), S9 (toluene, formaldehyde, benzene, alcohol, acetone), S10 (hydrogen), S11 (liquefied gas, alkanes), S12 (liquefied gas, methane), S13 (methane), and S14 (combustible gas, smoke). Each pomegranate sample was put into a 20 mL headspace vial, then incubated at 50 °C for 30 min. The following instrument settings were used: sample interval time of 1 s, sensor self-cleaning time of 40 s, sample preparation duration of 5 s, an injection velocity of 300 mL/min, and sample measurement time of 90 s.

### 2.3. E-Tongue Analysis

The taste properties of the pomegranate samples were tested using an electronic tongue (iTongue20, THINKSENSO, Zhejiang Zheke Instrument Equipment Co., Ltd., Hangzhou, China), which was composed of 6 taste sensors, including CA0, GL1, C00, AE1, AAE, and CT0 for sour, sweet, bitter, astringent, umami, and salty, respectively, and two reference electrodes. The samples were pretreated following the protocol outlined by the previous report [18] with some modifications. 5 g of each pomegranate sample was diluted with 20 mL of distilled water, thoroughly homogenized, and then the solution was centrifuged at 10,000 rpm for 15 min at 4 °C. Subsequently, the supernatant was subjected to filtration for further analysis. The detection time was set to 120 s, and before each measurement, the sensor was thoroughly rinsed with deionized water for 30 s to ensure accuracy.

### 2.4. HS-GC-IMS Analysis

VOCs in pomegranate samples were investigated using HS-GC-IMS (FlavourSpec^®^ flavor analyzer, G.A.S., Dortmund, Germany). A 5 g portion of each pomegranate sample was placed into a 20 mL headspace vial. The conditions for headspace injection were as follows: an incubation time of 15 min, an incubation temperature of 60 °C, a needle temperature of 65 °C, and an injection volume of 200 μL. For the GC conditions, a MAX-WAX chromatographic column (30 m × 0.53 mm × 1 μm, RESTEK Company, Bellefonte, PA, USA) was used, with N_2_ (purity ≥ 99.999%) as the carrier gas. Then the flow rate was set at 2 mL/min for the first 2 min, subsequently increased to 10 mL/min over 3 min, followed by a further increase to 100 mL/min over 20 min, maintaining this flow rate for 5 min. The IMS was operated under conditions with a drift tube temperature of 60 °C and a gas flow rate of 150 mL/min. Volatile compounds were identified by matching retention time (RT) and drift time against the National Institute of Standards and Technology (NIST) 2020 GC retention index database and IMS migration time databases, and the relative concentration of each compound was quantified by normalizing its peak areas to the signal intensity. Triplicate analyses were performed for each pomegranate sample.

### 2.5. HS-SPME-GC-MS Analysis

Pomegranate samples (5 g) were sealed in 20 mL headspace vials for subsequent analysis. Volatile compounds were extracted using a conditioned SPME extraction fiber exposed to the vial headspace for 50 min at room temperature, followed by thermal desorption in the GC inlet at 280 °C for 30 s. GC conditions were as follows: an HP-INNOWAX capillary column (60 m × 0.25 mm × 0.25 μm, Agilent Technologies, Santa Clara, CA, USA) was employed, with an inlet temperature set to 250 °C. Nitrogen (N_2_) (purity > 99.999%) was used as the carrier gas at a flow rate of 1 mL/min. Heating process conditions: the temperature started at 50 °C for 3 min, then increased to 100 °C at a rate of 5 °C/min for 5 min, followed by a rise to 180 °C at 10 °C/min for 5 min, and finally ramped to 220 °C at 10 °C/min for 15 min. Mass spectrometry conditions: electron ionization source, ionization voltage was set to 70 eV; ion source temperature at 230 °C; transmission line temperature at 250 °C; interface temperature at 250 °C; mass scanning range 29–500 *m*/*z*. Qualitative identification was performed using the NIST17 mass spectral library, with retention index (RI) verification via a C7–C40 n-alkane calibration and cross-referencing of the calculated RI values with the NIST Chemistry WebBook. Relative quantification was based on peak area normalization.

### 2.6. ROAV Analysis

ROAV analysis is a commonly used method to evaluate the contribution of VOCs to the overall odor profile of pomegranate samples. This approach integrates both the compound’s relative concentration and its odor detection threshold, thereby quantifying its sensory relevance [20]. Compounds with an ROAV greater than 1 are generally considered key aroma-active substances, exerting a significant influence on the overall aroma of pomegranate fruits. The ROAV is calculated according to the following equation:

$$ROAV\approx 100\times \frac{C_{i}}{T_{i}}\times \frac{T_{max}}{C_{max}}$$

where *C_i_* (mg/kg) represents the relative concentration of VOCs, and *T_i_* (mg/kg) denotes its odor thresholds in water. *C_max_* and *T_max_* correspond to the relative percentage content and odor threshold, respectively, of the compound contributing most strongly to the overall flavor profile of the sample.

### 2.7. Data Analysis

The findings were expressed as means ± standard deviation (SD), and statistical analyses were performed using IBM SPSS Statistics 27.0 (SPSS Inc., Chicago, IL, USA) to evaluate significant differences among samples. HS-GC-IMS data were processed using VOCal software v.0.4.03 (G.A.S., Dortmund, Germany) along with three supplementary plug-ins: Reporter, Gallery Plot, and Dynamic PCA. Data visualization was performed using Origin 2024 (Origin Lab Corporation, Northampton, MA, USA) and Chiplot (https://www.chiplot.online/ accessed on 10 September 2025). OPLS-DA, permutation test, and the predictor importance were conducted by SIMCA 14.1 (Umeå, Sweden).

## 3. Results and Discussion

### 3.1. E-Nose Analysis

**Figure 1:**
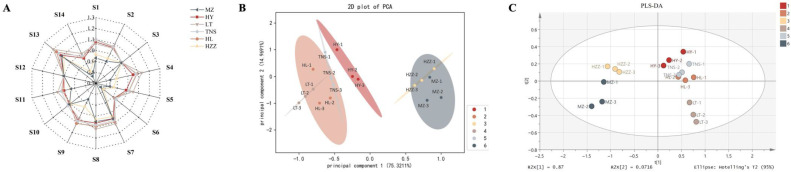
Analysis of pomegranate samples from different origins by electronic nose. (**A**) Radar plot of sensor response profiles. (**B**) PCA score plot. (**C**) PLS-DA score plot.

The E-nose system is sensitive to odors within its detection range, and reflects the overall aroma profiles of the samples to some extent [21]. In this study, an E-nose characterized the aroma profiles of six pomegranate samples, with the sensor response patterns from 14 metal oxide sensors visualized in the radar plot (Figure 1A). Distinct sensor response was observed for pomegranate samples of different origins, reflecting variations in the degree to which aroma substances from these pomegranates interact with the sensors. Among all sensors, the methane-sensitive sensor S13 showed the strongest responses across all pomegranate samples, particularly for LT and HL, suggesting elevated emissions of methane or related short-chain alkanes likely associated with metabolism during pomegranate ripening. In contrast, the sensors for aromatic compounds S6 exhibited the lowest responses among all samples, especially in HZZ and MZ, reflecting differences in flavor-related metabolites among pomegranates from different geographical origins. Furthermore, HL exhibited high response values across most sensors, excluding S10 and S12, whereas MZ showed universally low responses except for these same two sensors.

The PCA plot could effectively differentiate pomegranate samples from multiple origins based on E-nose sensor responses (Figure 1B), with the first two principal components (PC1 and PC2) explaining 90.29% of the total variance (PC1: 75.32%, PC2: 14.97%) and capturing the dominant aroma profiles. In the PCA space, MZ and HZZ formed a distant cluster significantly separated from other samples, reflecting their divergent aroma profiles and consistent with their sensor response patterns in the radar chart. While TNS and HL showed similar aroma characteristics, the overall profiles of pomegranate samples varied significantly by origin, enabling geographical differentiation based on these distinct aromatic patterns. Linear discriminant analysis (LDA) is a supervised dimensionality reduction method that enhances inter-class distinctions while reducing intra-class scatter [22]. As shown in Figure S1A, the LDA plot confirmed the PCA results, with LDA1 (70.2%) and LDA2 (23.8%) achieving clearer separation of pomegranate samples from different origins compared to PCA. In addition, the E-nose’s capability to discriminate pomegranate geographical origins was further validated using a PLS-DA model. As shown in Figure 1C, the model clearly separated pomegranate samples by origin based on their aroma profiles. Meanwhile, the variable importance analysis based on the PLS-DA model revealed S1, S2, S6, S9, and S13 as primary discriminators (VIP > 1), aligning with their response patterns in radar profiles Figure S1B. These results demonstrate the E-nose’s potential as a rapid, non-destructive method for preliminary origin discrimination of pomegranates; however, since it cannot identify specific compounds, complementary GC-based analyses are necessary.

### 3.2. E-Tongue Analysis

**Figure 2:**
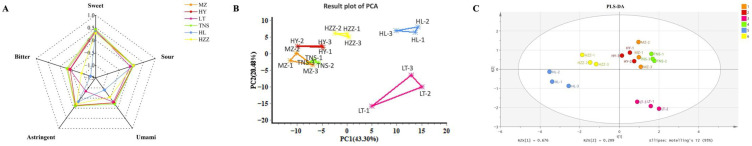
Analysis of pomegranate samples from different origins by electronic tongue. (**A**) Radar plot of sensor response profiles. (**B**) PCA score plot. (**C**) PLS-DA score plot.

In this study, an E-tongue was employed to evaluate taste variations among pomegranate samples from distinct regions. Notably, no salty taste response was detected in any pomegranate samples. Basic sensory profiles were visualized in a radar chart (Figure 2A), which highlighted that those responses to sweet and sour tastes were dominant flavors among all samples, aligning with pomegranate’s characteristic taste. The taste radar profiles revealed significant differences in taste attributes, and the signal strength differences of bitter, astringent, and umami were obvious. Specifically, HL showed the lowest values in bitter and umami responses, while LT exhibited minimal astringent among all samples. It is worth noting that the TNS samples displayed significantly higher sweetness and acidity than other samples, which may be attributed to the longer daily light exposure time during fruit maturation in their origin, Xinjiang. In addition, all pomegranate samples except LT maintained consistent taste attribute hierarchies, although with different response values. To further analyze taste differences, a PCA plot of the E-tongue data was constructed (Figure 2B), revealing clear segregation among samples, with PC1 (43.30%) and PC2 (20.48%) cumulatively explaining 63.78% of variance. Samples with smaller spatial distances showed more similar taste profiles, specifically, TNS, MZ, and HY samples clustered closely, suggesting similar taste attributes, whereas other samples formed distinct groups, reflecting their divergent taste characteristics, which is consistent with their taste response patterns in the radar chart.

The LDA plot demonstrated clear separation among the six pomegranate samples (discriminant index = 99.97), confirming significant taste profile differences among pomegranates from different origins (Figure S1C). In addition, the E-tongue’s capability to discriminate pomegranate geographical origins was further validated using a PLS-DA model. As shown in Figure 2C, the model clearly separated pomegranate samples by origin based on their taste profiles, although MZ and HY samples clustered closely. Meanwhile, the variable importance analysis based on the PLS-DA model revealed sour and sweet as primary discriminators (VIP > 1) (Figure S1D), aligning with their response patterns in radar profiles. These results demonstrate that the E-tongue provides critical insights into how regional factors shape pomegranate taste profiles. Moreover, the observed clustering pattern of MZ and HY samples may reflect similarities in cultivation practices or genetic factors. Interestingly, while taste analysis effectively differentiated samples, prior evidence suggests smell may be more decisive for geographic origin discrimination—a pattern consistent with findings in Chinese regional milk flavors from different regions of China [23], though further volatile compound analysis is needed to confirm this for pomegranates.

### 3.3. Volatile Compounds in Pomegranate from Different Origins Were Characterized via HS-GC-IMS

**Figure 3:**
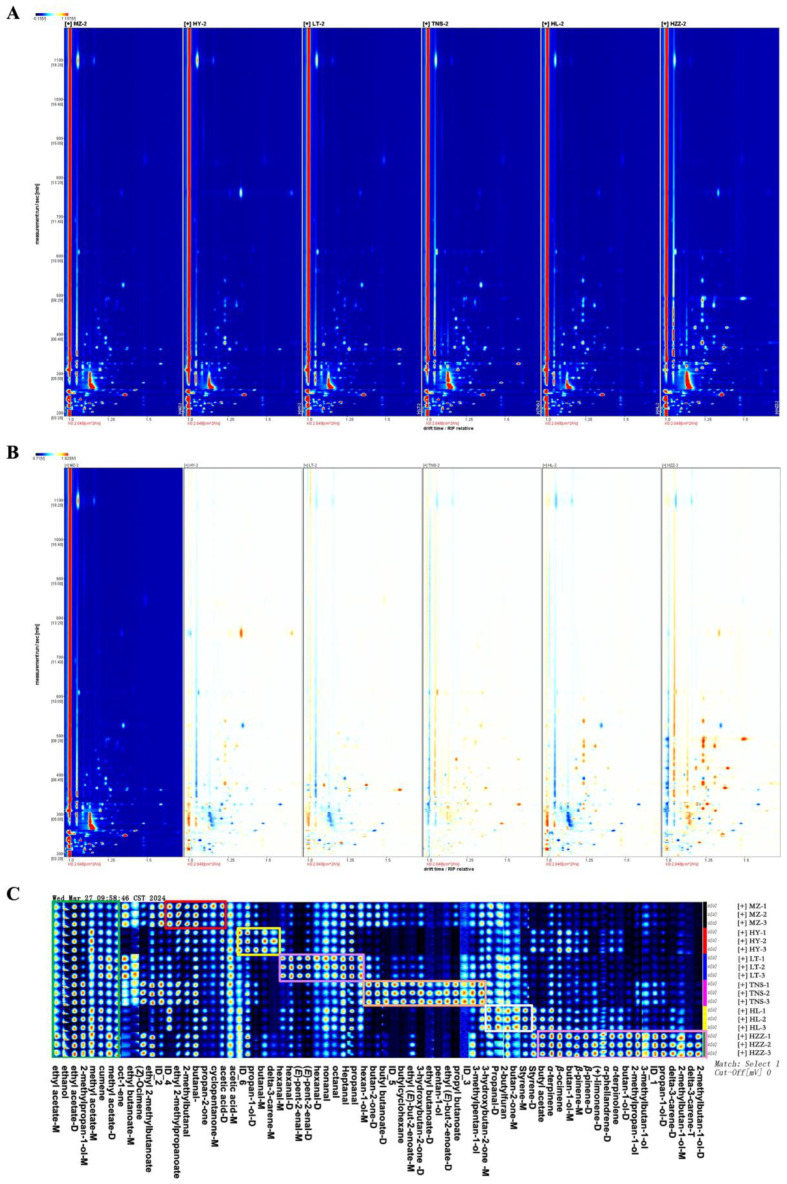
The information of VOCs profiles determined via HS-GC-IMS in pomegranate samples from different origins. (**A**) Two-dimensional topographic plots. (**B**) Difference comparison topographic plots. (**C**) Fingerprint of VOCs in pomegranate samples.

HS-GC-IMS was used to analyze the VOCs in pomegranate samples (MZ, HY, LT, TNS, HL, and HZZ) collected from six different regions. The two-dimensional (2D) topographical visualization (Figure 3A) revealed significant regional variations in VOC profiles, with the reactive ion peak (RIP) marked by a red vertical line at abscissa 1.0. VOCs were well-separated under specific conditions of retention time (RT: 100–500 s) and drift time (DT: 1–1.25 s), as evidenced by the distinct spatial distribution of compounds. A differential comparison model was generated using the MZ sample as a reference (Figure 3B), which highlighted pronounced variations in VOC concentrations, with red indicating higher concentrations and blue or white indicating lower concentrations. Additionally, some VOCs with high proton affinity were observed to form dimers or trimers during migration. The results clearly show significant differences in VOC profiles, particularly, the HL and HZZ samples exhibited the most pronounced dissimilarities with MZ. Although VOC types were generally similar across regions, the aroma fingerprints demonstrate significant concentration differences, underscoring the regional variability in pomegranate aroma profiles (Figure 3C). Additionally, some VOCs with high proton affinity were observed to form dimers or trimers during migration. The green rectangular box highlights VOCs common to all pomegranate samples, with minimal concentration variations, notably ethyl acetate, and these substances may form the foundation of pomegranate aroma. The red rectangular box highlights six compounds predominantly found in the MZ sample, including ethyl 2-methylpropanoate, 2-methylbutanal, butanal, propan-2-one, cyclopentanone, and acetic acid. The yellow rectangular box identifies four VOCs with higher concentrations in the HY samples, including propan-1-ol, butanal, delta-3-carene, and hexan-1-ol. Compared with other pomegranate samples, the main compounds in LT are aldehydes such as (*E*)-hex-2-enal and (*E*)-pent-2-enal, while in HZZ samples, the prominent compounds are alcohols and alkenes. Additionally, the TNS and HL sample groups were also marked in the figure with more prominent components than the other sample groups. In summary, these compounds can serve as characteristic markers to distinguish pomegranate samples of different geographical origins. Furthermore, it is noteworthy that sample HZZ maintained a considerable variety of characteristic volatile compounds despite exhibiting lower E-nose and E-tongue responses, a phenomenon also observed in studies of squid aroma profiles where sensor outputs did not fully reflect compound abundance due to low odor thresholds or sensor selectivity [24].

As indicated in Table 2, a total of 76 IMS signals were detected, of which 69 were identified by NIST and IMS databases, including 9 alcohols, 9 aldehydes, 9 esters, 4 ketones, 9 terpenes, 2 aromatic hydrocarbons, 1 acid, and 3 other compounds. Notably, a portion of these compounds was present in monomer (M) or dimer (D) forms, these represent the same chemical identity rather than distinct compounds. Alcohols, particularly ethanol and 2-methylpropan-1-ol, with a significant content in pomegranate, offer floral, sweet, and sour notes. These alcohols were identified as flavor contributors, consistent with previous studies that noted ethanol’s potential aroma contribution in pomegranate juice [25]. Esters, particularly ethyl acetate, also play an important role in contributing to the pleasant aroma of pomegranate, as previously reported [26]. Similarly, terpenes, such as (+)-limonene, delta-3-carene, *β*-pinene, *β*-ocimene, and *α*-phellandrene, were predominant across samples, aligning with earlier findings [9]. Notably, *α*-phellandrene has a black pepper and mint-like aroma and was identified as a characteristic aroma compound of *Citri Reticulatae Pericarpium*, but in previous studies, it was considered a general volatile aroma substance of pomegranate [27]. In addition, aldehydes are important VOCs with unique flavor characteristics, particularly hexanal, which were significant contributors to the flavor, with grassy notes at low concentrations and nutty, fatty aromas at higher levels, as discussed by [28] and supported by previous studies on pomegranate [29,30].

**Figure 4:**
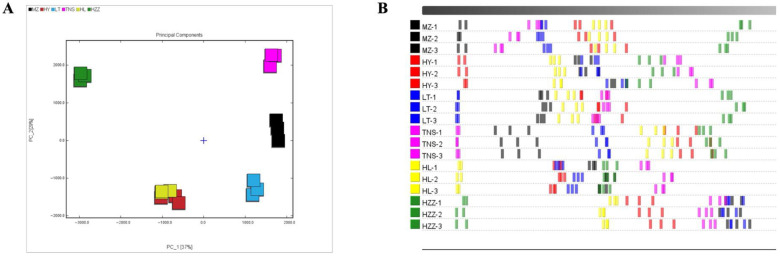
Analysis of pomegranate samples from different origins via HS-GC-IMS. (**A**) PCA score plot. (**B**) Euclidean distance diagram.

**Figure 5:**
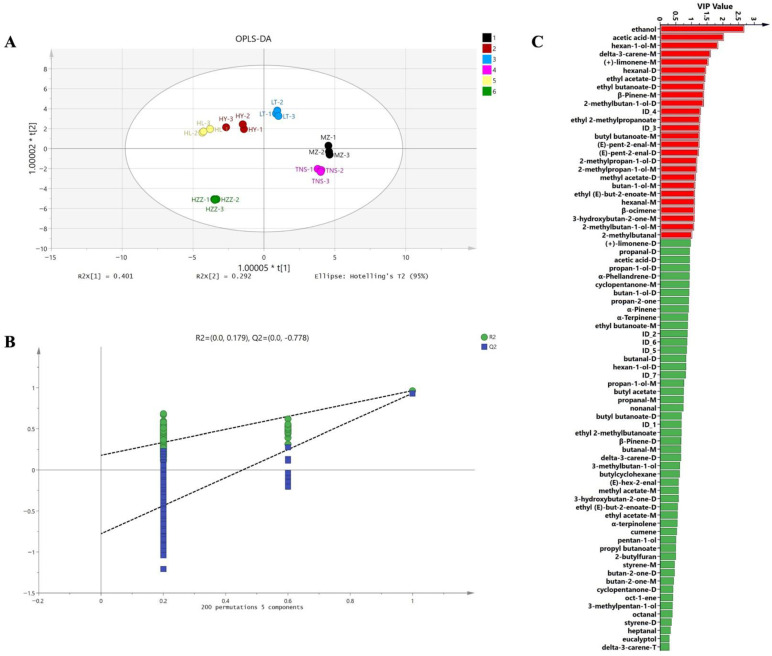
Analysis of pomegranate samples from different origins via HS-GC-IMS. (**A**) OPLS-DA score plot. (**B**) Displacement verification diagram (permutation tests (*n* = 200)). (**C**) VIP value plot. Red bar (VIP > 1) indicates that the compound has important differences among pomegranate origins.

The PCA plot revealed distinct separation patterns among pomegranate samples, with PC1 (37%) and PC2 (29%) collectively explaining 66% of the total variance, demonstrating effective dimensionality reduction that preserved major compositional variability (Figure 4A). While most pomegranate samples demonstrated distinct separation, indicating significant inter-sample differences, HL and HY samples formed a tight cluster, suggesting similar aroma profiles. Additionally, HZZ exhibited marked isolation from all other samples, reflecting its unique VOCs signature. The Euclidean distance analysis demonstrated that a shorter inter-group distance indicates smaller differences among samples, while a shorter intra-group distance reflects better parallelism within the sample group [31]. The pomegranate samples MZ and TNS showed were close in distance, indicating higher similarity compared to other samples (Figure 4B). Similarly, HY and HL demonstrated proximity, and this pattern aligned with their tight clustering in the PCA plot. Additionally, the small within-group distances demonstrated good experimental repeatability across all samples. To characterize flavor profiles across geographical origins, an OPLS-DA model was constructed using 46 common volatile compounds as the dependent variable and origin categories as the independent variables. The model effectively explained 99.0% of the total observed variability among six pomegranate samples from different origins, demonstrating excellent model fit (R2X = 0.980, R2Y = 0.985), and predictive reliability (Q2 = 0.972). The score plot (Figure 5A) showed complete separation among the six pomegranate samples from different origins with tight intra-group clustering, demonstrating the model’s discriminative capacity. Permutation testing (*n* = 200) further confirmed model validity, with intercept values of R2 = (0.0, 0.179), Q2 = (0.0, −0.778), where the negative Q2 indicated no overfitting, and the model verification is valid (Figure 5B). These results validate the model’s applicability for pomegranate origin authentication. Furthermore, the key volatile compounds contributing to geographical discrimination of pomegranate samples were selected via VIP analysis, with a threshold of VIP > 1 serving as the screening criterion [32]. As shown in Figure 5C, there are 22 key compounds with VIP value of >1, comprising 20 identified compounds: ethanol, acetic acid, hexan-1-ol, delta-3-carene, (+)-limonene, hexanal, ethyl acetate, ethyl butanoate, *β*-pinene, 2-methylbutan-1-ol, ethyl 2-methylpropanoate, butyl butanoate, (*E*)-pent-2-enal, 2-methylpropan-1-ol, methyl acetate, butan-1-ol, ethyl (*E*)-but-2-enoate, β-ocimene, 3-hydroxybutan-2-one, and 2-methylbutanal, while 2 unknown compounds (ID_4 and ID_3). These compounds account for the major differences in volatile composition across the geographic origins.

### 3.4. Volatile Compounds in Pomegranate from Different Origins Were Characterized via HS-SPME-GC-MS

**Figure 6:**
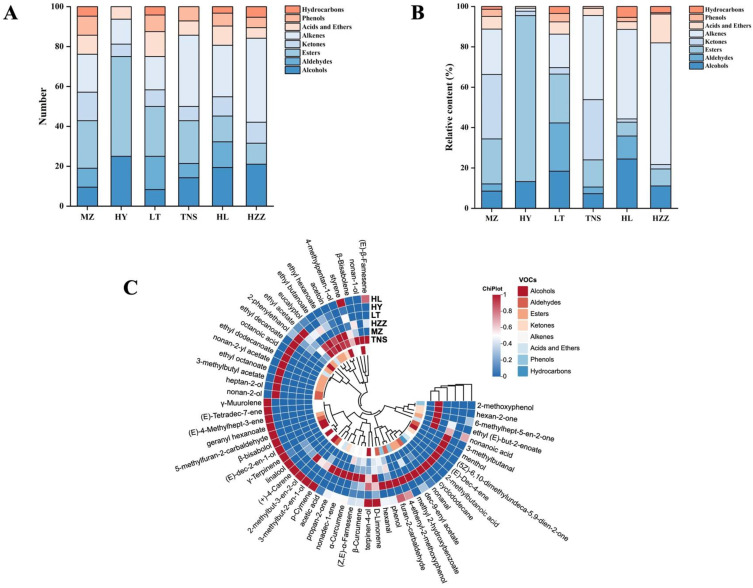
Analysis of pomegranate samples from different origins via HS-SPME-GC-MS. (**A**,**B**) Percentage stacked histogram. (**C**) Hierarchical clustering heat map: the color indicates the concentration of the compound, and the blue and red indicate the low and high concentration, respectively.

The volatile compounds in pomegranate samples from different origins were analyzed using HS-SPME-GC-MS. A total of 58 VOCs were identified, comprising 12 alcohols, 5 aldehydes, 12 esters, 5 ketones, 14 alkenes, 5 acids and ethers, 3 phenols, and 2 hydrocarbons (Table 3). For clearer comparison, the distribution of VOCs across chemical classes in each pomegranate sample is shown in Figure 6A. Compared with other pomegranate samples, the HL sample contained the greatest number of alkenes (12), while HY had the most esters (7). Notably, no aldehydes, phenols, or hydrocarbons were identified in HY, and HZZ similarly lacked aldehydes, and TNS showed no acids or hydrocarbons. As shown in Figure 6B, the relative abundances of VOC varied significantly among pomegranate samples, where alkenes and esters showed relatively higher concentrations and were the main aroma components of pomegranate, aligning with previous reports [9].

Hierarchical cluster analysis was performed to further characterize these differences, revealing significant variations in VOC profiles among pomegranate origins (Figure 6C). Alkene compounds, particularly terpenes, which typically possess floral and fruity aroma profiles, have been demonstrated to play a critical role in modulating pomegranate flavor and significantly influence consumer preference [33]. (*Z*, *Z*)-*α*-Farnesene and limonene were identified as the predominant volatile compounds in pomegranate juice [30,34], while *γ*-terpinene, (+)-4-carene, (*E*)-.*β*.-Famesene, and *β*-curcumene constitute key components responsible for the characteristic fruity and fresh aroma of pomegranate [12,35]. Similarly, these reported alkenes were detected in our pomegranate samples, displaying distinct distribution patterns with significantly higher accumulation in HZZ, which facilitates origin differentiation.

Notably, *α*-phellandrene and *β*-farnesene, previously suggested to play a significant role in shaping the overall aroma profile of pomegranate juice [34], were absent in this study, possibly due to differences in the sources of pomegranates used. Ester compounds are formed through the reaction between organic acids and alcohols during fruit ripening, which significantly contributes to the development of fruity aromas and flavors. Moreover, ethyl acetate, characterized by its sweet, fruity odor, is a commonly reported aroma component in various fruits and vegetables, even has also been reported as the predominant component in pomegranate juice [26,36]. Similarly, this study found that ethyl acetate is the main ester substance in all pomegranate samples, while distinctive ester compositions were observed in all samples except TNS. Notably, some esters, such as oct-1-en-3-yl acetate, have been previously reported as the primary odorant in pomegranate (cv. Ganesh) from India, but this compound was not detected in our study [9], it can be seen that there are differences in VOCs of pomegranate from different geographical sources.

In addition, alcohols have also made significant contributions to the aroma composition of pomegranate. Previous studies have identified α-terpineol as the predominant aromatic compound in pomegranate juices with a floral/lilac odor [26,29,34]. Moreover, terpinen-4-ol (sweet/grassy) is considered a key aroma compound of pomegranate juice, which was also identified in our study. Furthermore, the floral fragrance is a crucial component of pomegranate’s overall aroma, with linalool appearing to play a central role in defining this floral note [35], though it was only identified in HL and HZZ samples. Similarly, nonan-2-ol, characterized by fruity green or fatty odor, was only detected in the HY sample, whereas nonan-1-ol with a grassy aroma was uniquely present in the TNS sample. It appears that aldehydes also contribute to the overall aroma of fruits, and all aldehydes identified in this study were present in the HL sample, except for 3-methylbutanal, whereas the HY sample lacked 5-methylfuran-2-carbaldehyde. Hexanal, known for its characteristic green note, was determined to be a vital aroma-active compound in pomegranate [37] and was detected in all samples in this study except HY and HZZ. In addition, we found that acetoin, the most abundant ketone, characterized by its strong fatty, creamy, and buttery scent and previously documented as an aroma constituent in pomegranate juice [26], was undetected in the HL sample. Overall, the VOCs of pomegranate are highly complex, with various compounds contributing to its characteristic flavor. Notably, significant variations in the relative content of these VOCs are observed in pomegranate samples from different geographic regions, primarily reflected in the esters, terpenes, as well as alcohols.

**Figure 7:**
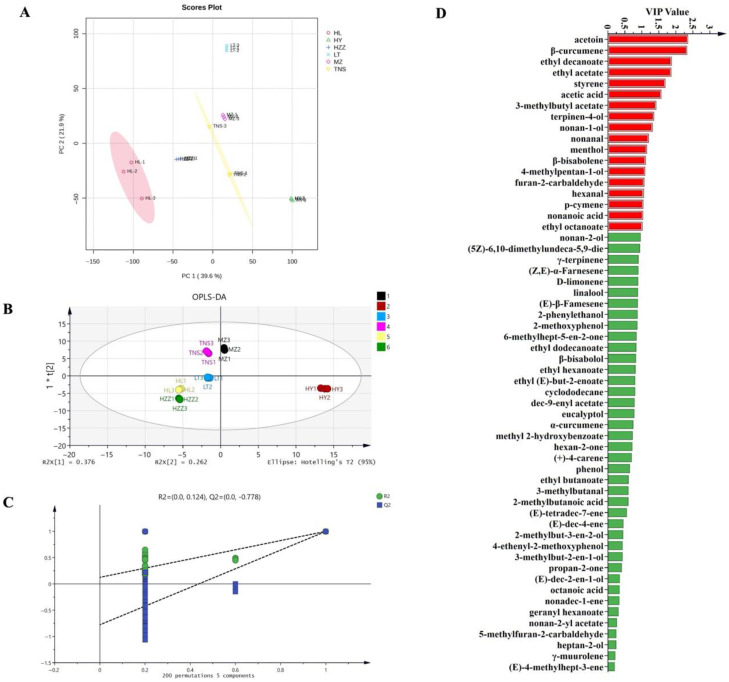
Analysis of pomegranate samples from different origins via HS-SPME-GC-MS. (**A**) PCA score plot. (**B**) OPLS-DA score plot. (**C**) Displacement verification diagram (permutation tests (n = 200)) (D) VIP value plot. The red bar (VIP > 1) indicates that the compound has important differences among pomegranate origins.

The PCA plot derived from HS-SPME-GC-MS exhibited a more distinct separation between pomegranate samples (Figure 7A) compared to the tightly clustered HL-HY group observed via HS-GC-IMS. Conversely, the supervised OPLS-DA model (Figure 7B) score plot demonstrated only partial separation, whereas HS-GC-IMS achieved superior discrimination of pomegranate origins. These findings indicate that the different analytical assays yielded varying results in detecting volatile compounds. Permutation tests further confirmed the OPLS-DA model’s validity (Figure 7C) and there are 18 key compounds with VIP value of > 1 (Figure 7D). Among them, acetoin (buttery/creamy), *β*-curcumene (woody), ethyl acetate (fruity), terpinen-4-ol (herbal–woody notes), and nonan-1-ol (floral/sweet notes), and hexanal (green) were both characteristic volatile compounds of pomegranate [35,37], these compounds could be used to distinguish pomegranate samples from different origins. These findings suggest that the identified VOCs can serve as potential markers for differentiating pomegranates from various origins.

### 3.5. Key Aroma Substances Analysis in Pomegranate from Different Origins

Given that the composition of VOCs does not always directly correspond to the perceived flavor, ROAV analysis provides a reliable approach for identifying key aroma-active compounds contributing to the overall aroma profile [38]. Compounds with an ROAV greater than one were considered key contributors shaping the characteristic flavor profile of pomegranate. Specifically, compounds with 0.1 ≤ ROAV < 1.0 showed a slight modifying effect, those with 1.0 ≤ ROAV < 10.0 made a significant contribution, and those with 10.0 ≤ ROAV < 100.0 exerted a decisive influence on the overall aroma profile [39].

ROAVs of volatile aroma compounds detected via HS-GC-IMS and HS-SPME-GC-MS were calculated based on their odor thresholds and odor descriptions to identify key aroma-active compounds (ROAV ≥ 1) in six pomegranate samples. According to Table 4, 22, 22, 27, 20, 21, and 13 VOCs with ROAV ≥ 1 were detected in MZ, HY, LT, TNS, HL, and HZZ, respectively. Furthermore, 23 aroma-active compounds were shared across all pomegranate samples, whereas in HY and TNS, only a few compounds exhibited ROAV values below 0.1, excluding compounds not detected. All compounds identified via HS-GC-IMS were consistently detected across every pomegranate sample. In contrast, HS-SPME-GC-MS analysis revealed that only ethyl acetate, styrene, and D-limonene were found in all samples, whereas the presence of other compounds differed by region (Table 4). For example, nonan-1-ol was detected exclusively in TNS, 3-methylbutyl acetate, and 3-methylbutanal only in HY, and 2-methoxyphenol solely in MZ. Moreover, (*Z*, *E*)-*α*-Farnesene was absent only in HY, whereas acetoin was not detected in HL. The ROAV of all 17 compounds, including nonanal, ethyl 2-methylbutanoate, ethyl 2-methylpropionate, 2-methylbutanal, oct-1-ene, butanal, 3-methylbutanal, ethyl acetate, ethyl butanoate, ethyl hexanoate, ethyl decanoate, acetoin, D-limonene, styrene, and 2-methoxyphenol, exceeded 10. Specifically, ethyl 2-methylbutanoate, ethyl 2-methylpropionate, ethyl acetate, ethyl decanoate, acetoin, and 2-methoxyphenol each reached an ROAV of 100.

**Figure 8:**
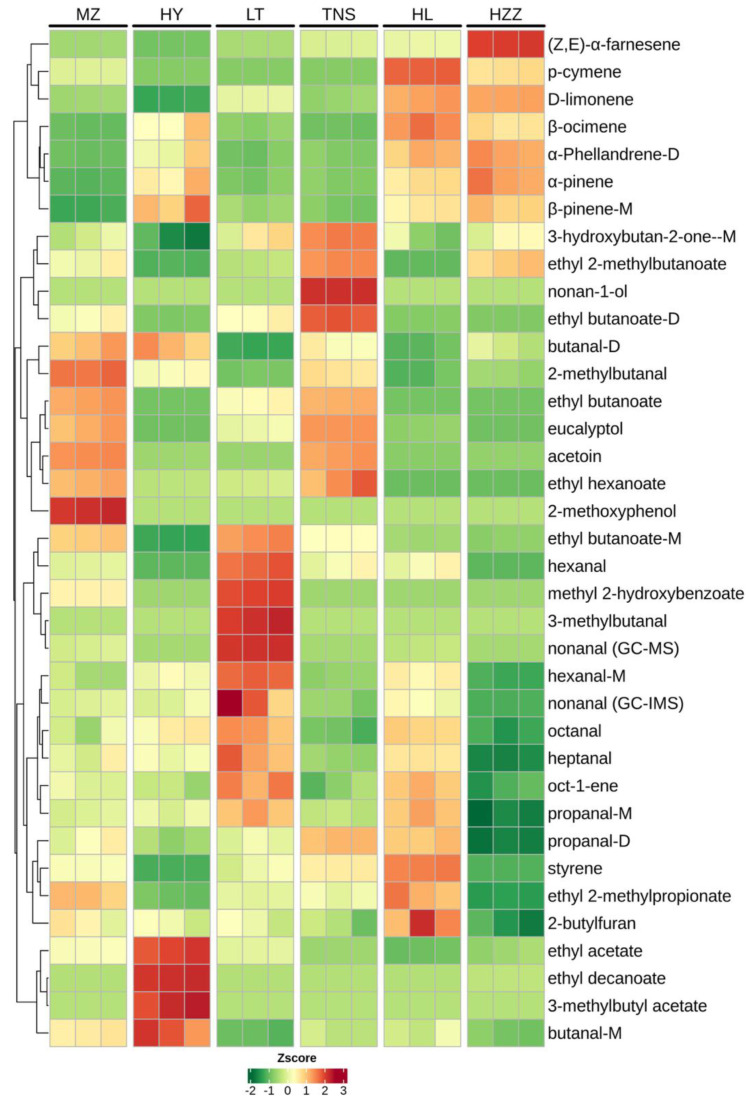
Heatmap of volatile compounds (ROAV > 1) in pomegranate samples from different origins via HS-GC-IMS and HS-SPME-GC-MS.

The heatmap revealed that 2-methylbutanal and 2-methoxyphenol accounted for a significant proportion in the MZ sample, being associated with almond, nut, fermented, and wood aromas (Figure 8). In contrast, the HY sample was characterized by elevated levels of ethyl acetate, ethyl decanoate, 3-methylbutyl acetate, and butanal, contributing to a pronounced fruit profile. In the LT sample, 3-methylbutanal, methyl 2-hydroxybenzoate, and nonanal were abundant, representing citrus and green odorants. The TNS sample exhibited higher concentrations of floral, creamy, green, and fruit compounds such as nonan-1-ol, ethyl butanoate, and ethyl hexanoate. The HL sample was dominated by distinctively concentrated floral, citrus, and fresh components, which may serve as differentiating aroma-active compounds among the samples. The HZZ sample was primarily associated with floral and woody notes. Overall, the results indicate that HY and TNS are similarly fruit-forward, LT and HL share citrus–green, floral, fresh notes, and MZ and HZZ both show woody/phenolic characteristics.

### 3.6. Comparison of the Ability of HS-GC-IMS and HS-SPME-GC-MS to Identify of Main Biomarkers and Aroma Characteristics of Pomegranate from Different Origins

**Figure 9:**
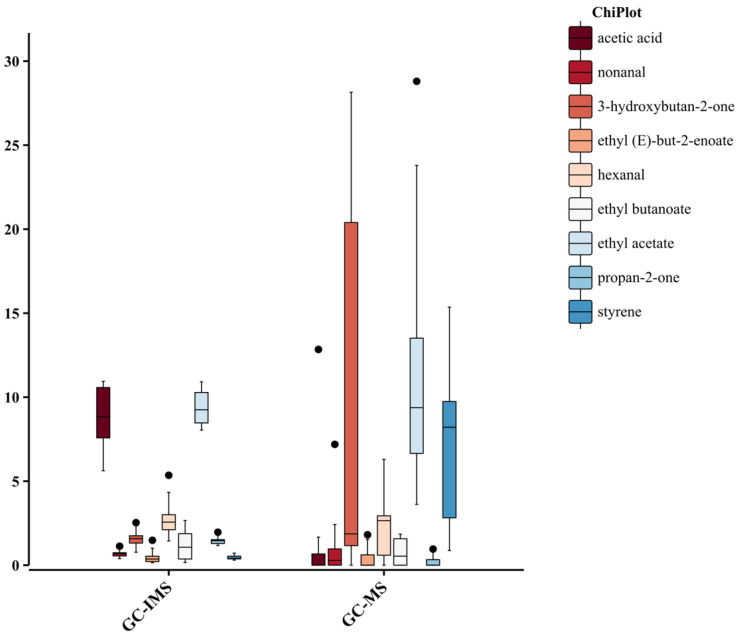
The Boxplots of relative abundances for nine VOCs detected via HS-GC-IMS and HS-SPME-GC-MS.

HS-GC-IMS and HS-SPME-GC-MS were employed to characterize volatile profiles of pomegranates from different origins. According to the previous analyses, HS-GC-IMS demonstrated the ability to discriminate between pomegranates of different origins, while HS-SPME-GC-MS showed better clustering ability for pomegranates of different origins. As shown in Figure S2. 46 and 58 volatile compounds were identified in pomegranate via HS-GC-IMS and HS-SPME-GC-MS, respectively. Aldehydes were more frequently detected via HS-GC-IMS, whereas other chemical classes were more readily detected via HS-SPME-GC-MS. Only nine compounds were shared between the two platforms, indicating limited overlap and methodological complementarity. For these shared compounds, cross-platform boxplots revealed clear method-dependent differences (Figure 9). HS-GC-IMS showed higher central tendencies with narrower dispersion for short-chain polar compounds (e.g., acetic acid and ethyl acetate), whereas HS-SPME-GC-MS yielded higher medians and broader spreads for 3-hydroxybutan-2-one (acetoin) and styrene. The remaining nonanal, hexanal, propan-2-one, ethyl butanoate, ethyl (*E*)-but-2-enoate) generally displayed low abundances on both platforms with occasional outliers. These patterns are consistent with platform-specific selectivity and support integrating both methods to achieve a more comprehensive characterization of pomegranate volatile compounds.

**Figure 10:**
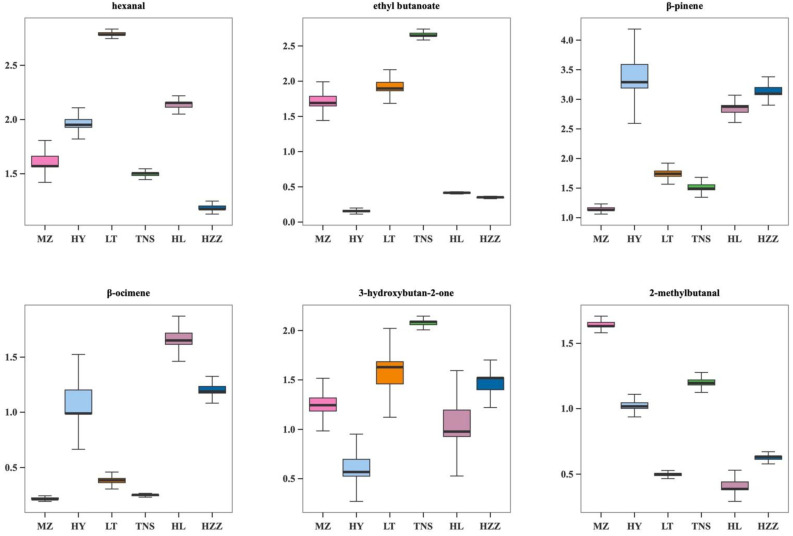
The box plot distribution of the principal aroma components in pomegranate samples from different origins via HS-GC-IMS.

Furthermore, integrating the VIP values (VIP > 1) from two OPLS-DA models, additional compounds were identified as potential key contributors to the differentiation of pomegranate samples from different origins. Specifically, Figure 10 presents the box plots of hexanal, ethyl butanoate, *β*-pinene, *β*-ocimene, 3-hydroxybutan-2-one and 2-methybutanal. Significant differences in these biomarkers detected via HS-GC-IMS were observed among the six pomegranate samples from different origins. The relative levels of ethyl butanoate and 3-hydroxybutan-2-one in the TNS sample were notably higher compared with the other samples. These compounds may serve as essential biomarkers for this particular group, and their odor descriptors (creamy and fruity) explain the characteristic aroma profile of TNS. In HL, *β*-ocimene was identified as a key biomarker, suggesting more pronounced fruity, sweet, and floral flavor notes. Moreover, hexanal was present at higher levels than others, corresponding to apple-like, fatty, fresh, and green notes, while *β*-pinene was recognized as a vital biomarker, with strong pine, polish, and woody aroma. Overall, HS-GC-IMS identified hexanal, ethyl butanoate, *β*-pinene, *β*-ocimene, 3-hydroxybutan-2-one, and 2-methylbutanal as the primary biomarkers for differentiating pomegranate samples from different origins.

**Figure 11:**
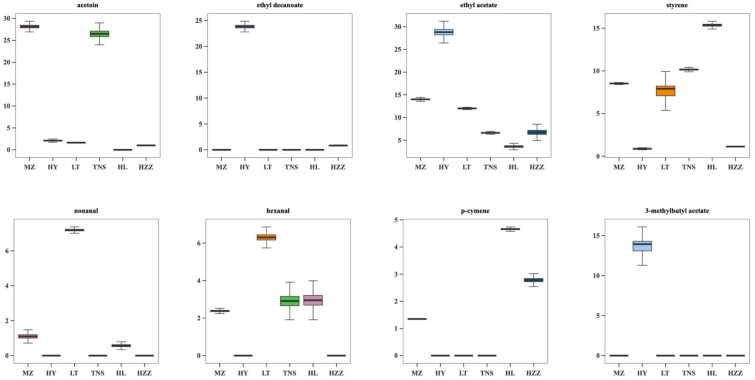
The box plot distribution of the principal aroma components in pomegranate samples from different origins via HS-SPME-GC-MS.

Figure 11 illustrates the distribution of eight key aroma compounds identified using the OPLS-DA model of HS-SPME-GC-MS, revealing distinct patterns among the six pomegranate samples. Compared with other samples, acetoin was present at markedly higher levels in MZ and TNS, conferring floral, green, fatty, buttery, and creamy notes. By contrast, HY was characterized by elevated concentrations of ethyl decanoate, ethyl acetate, and 3-methybutyl acetate, which are associated with fruity aromas such as grape, apple, and banana, suggesting that HY possessed a pronounced fruity profile. Similarly, LT exhibited higher levels of nonanal and hexanal, corresponding to floral, apple-, green-, and citrus- odors, indicating a more complex aroma profile. In comparison, HL contained a higher level of styrene, imparting aromatic nuances. Unlike these samples, HZZ did not show a dominance of any single compound. Notably, 3-hydroxybutan-2-one (acetoin) was detected via both HS-GC-IMS and HS-SPME-GC-MS, but its relative abundance exhibited certain differences between the two methods, possibly due to differences in extraction efficiency, ionization behavior, or sensitivity to matrix interferences. Overall, these findings demonstrate that pomegranate samples from different origins possess unique volatile signatures, with specific compounds serving as biomarkers of geographical origin.

## 4. Conclusions

This study comprehensively characterized the volatile profiles of pomegranates from six geographical origins using an integrated analytical approach combining intelligent sensory technologies (E-nose and E-tongue) and advanced chromatographic techniques (HS-GC-IMS and HS-SPME-GC-MS), complemented by multivariate statistical analysis. The results demonstrated that both the E-nose and E-tongue effectively distinguish pomegranate samples, with aroma profiles exhibiting greater discriminative power than taste characteristics. HS-GC-IMS analysis identified 46 volatile compounds in the pomegranate samples, with distinct flavor fingerprints observed for each geographical origin. 58 volatile compounds were identified via HS-SPME-GC-MS, and hierarchical clustering revealed distinct accumulation patterns of alkenes, esters, and alcohols among different geographical origins, demonstrating the potential of these characteristic volatiles as reliable markers to identify pomegranate origins. Subsequent OPLS-DA modeling coupled with VIP analysis (threshold > 1.0) screened 20 and 18 discriminant markers responsible for inter-origin variation, respectively. Notably, HS-GC-IMS exhibited superior discriminatory power in OPLS-DA modeling compared with HS-SPME-GC-MS, underscoring its effectiveness for origin authentication. ROAV analysis further revealed 14 key aroma-active compounds, including hexanal, nonanal, *β*-pinene, 3-hydroxybutan-2-one, and *β*-ocimene as potential regional markers. Collectively, these findings demonstrate that pomegranates from different geographical origins possess unique volatile signatures, with specific compounds serving as reliable biomarkers for authenticating origin and differentiating products. This work provides a scientific basis for the geographical traceability of pomegranates and offers guidance for quality control and product development in the pomegranate industry.

## Figures and Tables

**Table 1:** The information about pomegranate samples from different production regions.

Sample Number	Sample Producing Area	Altitude	Latitude	Longitude
MZ	Mengzi, Yunnan	1300–1800 m	23°15′~23°34′	103°19′~103°31′
HY	Huaiyuan, Anhui	≤330 m	32°43′~33°19′	116°45′~117°09′
LT	Lintong, Shanxi	400–1000 m	34°16′~34°44′	109°5′~109°27′
TNS	Hetian, Xinjiang	≤1200 m	36°99′~37°01′	80°73′~80°88′
HL	Huili, Sichuan	1000–1800 m	26°5′~27°12′	101°52′~102°38′
HZZ	Zaozhuang, Shandong	500–700 m	34°35′~34°51′	117°22′~117°49′

**Table 2:** Relative contents and odor descriptions of volatile compounds in pomegranates from different origins analyzed via HS-GC-IMS.

Compounds	CAS#	RI	Dt [a.u.]	Relative Content (%)						Odor Description *
				MZ	HY	LT	TNS	HL	HZZ	
acetic acid-M	C64197	1489	1.05459	9.66 ± 0.72 ^ab^	9.89 ± 0.68 ^a^	8.7 ± 0.15 ^bc^	6.79 ± 0.18 ^d^	7.62 ± 0.29 ^cd^	5.25 ± 0.05 ^e^	acid, fruit
acetic acid-D	C64197	1489	1.15933	1.26 ± 0.32 ^a^	1.05 ± 0.14 ^ab^	0.83 ± 0.02 ^bc^	0.6 ± 0.08 ^cd^	0.52 ± 0.05 ^cd^	0.37 ± 0.05 ^e^	acid, fruit
nonanal	C124196	1402.2	1.48852	0.67 ± 0.02 ^b^	0.69 ± 0.04 ^b^	1.13 ± 0.22 ^a^	0.51 ± 0.04 ^bc^	0.76 ± 0.04 ^b^	0.39 ± 0.00 ^c^	floral, green, lemon
hexan-1-ol-M	C111273	1367.8	1.3359	0.57 ± 0.08 ^c^	3.84 ± 0.52 ^a^	0.9 ± 0.31 ^bc^	0.46 ± 0.03 ^c^	0.86 ± 0.06 ^bc^	1.32 ± 0.05 ^b^	flower, grass, herb
hexan-1-ol-D	C111273	1367.8	1.66154	0.13 ± 0.03 ^b^	0.77 ± 0.16 ^a^	0.15 ± 0.02 ^b^	0.08 ± 0.01 ^b^	0.13 ± 0.03 ^b^	0.18 ± 0.02 ^b^	flower, grass, herb
3-hydroxybutan-2-one-M	C513860	1296	1.06057	1.25 ± 0.13 ^b^	0.63 ± 0.18 ^c^	1.55 ± 0.23 ^b^	2.07 ± 0.04 ^a^	1.09 ± 0.28 ^bc^	1.44 ± 0.14 ^b^	butter, creamy
3-hydroxybutan-2-one-D	C513860	1295.8	1.35071	0.2 ± 0.01 ^bc^	0.14 ± 0.00 ^d^	0.22 ± 0.02 ^bc^	0.46 ± 0.03 ^a^	0.18 ± 0.03 ^bc^	0.26 ± 0.06 ^b^	butter, creamy
butyl butanoate-M	C109217	1240.1	1.34511	1.56 ± 0.15 ^b^	0.39 ± 0.20 ^c^	1.71 ± 0.12 ^ab^	2.03 ± 0.10 ^a^	0.43 ± 0.06 ^c^	0.23 ± 0.03 ^c^	floral
butyl butanoate-D	C109217	1241.1	1.8405	0.22 ± 0.05 ^b^	0.09 ± 0.01 ^c^	0.26 ± 0.04 ^b^	0.51 ± 0.07 ^a^	0.1 ± 0.01 ^c^	0.06 ± 0.01 ^c^	floral
3-methylbutan-1-ol	C123513	1215.1	1.25243	0.4 ± 0.06 ^bc^	0.31 ± 0.08 ^cd^	0.19 ± 0.01 ^d^	0.5 ± 0.04 ^b^	0.45 ± 0.01 ^b^	0.78 ± 0.04 ^a^	burnt, floral
(+)-limonene-M	C138863	1205.6	1.23244	0.82 ± 0.03 ^e^	2.01 ± 0.25 ^c^	1.32 ± 0.09 ^d^	1.13 ± 0.06 ^de^	3.14 ± 0.14 ^b^	4.11 ± 0.12 ^a^	citrus
(+)-limonene-D	C138863	1205	1.3072	0.7 ± 0.04 ^d^	1.63 ± 0.13 ^b^	1.05 ± 0.07 ^c^	1.13 ± 0.01 ^c^	1.81 ± 0.11 ^ab^	1.96 ± 0.02 ^a^	citrus
(*Z*)-Ocimene	C3338554	1195.3	1.29581	0.09 ± 0.01 ^b^	0.04 ± 0.00 ^c^	0.13 ± 0.01 ^a^	0.1 ± 0.01 ^b^	0.04 ± 0.0 ^c^	0.04 ± 0.01 ^c^	floral, grass
cumene	C98828	1192.8	1.16786	0.37 ± 0.02 ^d^	0.45 ± 0.03 ^c^	0.57 ± 0.02 ^b^	0.38 ± 0.00 ^d^	0.64 ± 0.01 ^a^	0.29 ± 0.00 ^e^	-
heptanal	C111717	1194.2	1.33794	0.18 ± 0.02 ^b^	0.18 ± 0.01 ^b^	0.24 ± 0.02 ^a^	0.13 ± 0.00 ^c^	0.2 ± 0.00 ^b^	0.08 ± 0.00 ^d^	citrus, fat, green
ethyl (*E*)-but-2-enoate-M	C623701	1173.2	1.18502	0.46 ± 0.07 ^b^	0.17 ± 0.01 ^c^	0.48 ± 0.02 ^b^	1.22 ± 0.14 ^a^	0.18 ± 0.01 ^c^	0.12 ± 0.00 ^c^	tropical fruit
ethyl (*E*)-but-2-enoate-D	C623701	1173.4	1.58227	0.05 ± 0.01 ^b^	0.04 ± 0.01 ^b^	0.06 ± 0.00 ^b^	0.26 ± 0.07 ^a^	0.04 ± 0.00 ^b^	0.03 ± 0.01 ^b^	tropical fruit
*α*-phellandrene-D	C99832	1169	1.23339	0.33 ± 0.01 ^c^	0.96 ± 0.23 ^b^	0.37 ± 0.05 ^c^	0.42 ± 0.03 ^c^	1.27 ± 0.09 ^a^	1.38 ± 0.07 ^a^	citrus, fresh, mint, pepper, wood
butan-1-ol-D	C71363	1152.2	1.39021	0.11 ± 0.00 ^de^	0.08 ± 0.00 ^e^	0.14 ± 0.02 ^d^	0.24 ± 0.02 ^c^	0.48 ± 0.02 ^b^	1.17 ± 0.02 ^a^	-
butan-1-ol-M	C71363	1152	1.18658	0.92 ± 0.02 ^e^	0.99 ± 0.02 ^e^	1.29 ± 0.06 ^c^	1.16 ± 0.03 ^d^	2.3 ± 0.03 ^a^	1.69 ± 0.01 ^b^	-
ID_1	unidentified	1152.5	1.26304	0.22 ± 0.02 ^d^	0.17 ± 0.01 ^e^	0.23 ± 0.01 ^d^	0.43 ± 0.02 ^b^	0.27 ± 0.02 ^c^	0.76 ± 0.01 ^a^	-
cyclopentanone-M	C120923	1136.7	1.11246	0.66 ± 0.19 ^ab^	0.58 ± 0.08 ^ab^	0.4 ± 0.03 ^bc^	0.21 ± 0.02 ^c^	0.75 ± 0.20 ^a^	0.14 ± 0.02 ^c^	mint, cool
cyclopentanone-D	C120923	1137	1.3434	0.12 ± 0.07 ^a^	0.06 ± 0.01 ^ab^	0.03 ± 0.00 ^ab^	0.03 ± 0.01 ^b^	0.09 ± 0.03 ^ab^	0.03 ± 0.00 ^b^	mint, cool
2-butylfuran	C4466244	1136.7	1.1936	0.44 ± 0.03 ^b^	0.41 ± 0.02 ^b^	0.41 ± 0.03 ^b^	0.36 ± 0.03 ^bc^	0.54 ± 0.05 ^a^	0.29 ± 0.03 ^c^	wet hay
delta-3-carene-M	C13466789	1127.6	1.23417	1.55 ± 0.03 ^b^	2.84 ± 0.81 ^a^	0.58 ± 0.05 ^cd^	1.34 ± 0.10 ^bc^	0.4 ± 0.07 ^d^	0.53 ± 0.06 ^cd^	lemon
delta-3-carene-D	C13466789	1130.8	1.3091	0.11 ± 0.00 ^b^	0.1 ± 0.01 ^b^	0.15 ± 0.02 ^b^	0.15 ± 0.01 ^b^	0.48 ± 0.02 ^a^	0.58 ± 0.15 ^a^	lemon
delta-3-carene-T	C13466789	1131	1.76814	0.06 ± 0.01 ^b^	0.09 ± 0.02 ^b^	0.06 ± 0.01 ^b^	0.06 ± 0.01 ^b^	0.09 ± 0.01 ^b^	0.18 ± 0.07 ^a^	lemon
*β*-Pinene-M	C127913	1115	1.23183	1.15 ± 0.05 ^d^	3.42 ± 0.41 ^a^	1.74 ± 0.09 ^c^	1.52 ± 0.09 ^cd^	2.83 ± 0.12 ^b^	3.16 ± 0.13 ^ab^	pine, polish, wood
*β*-Pinene-D	C127913	1115.2	1.30639	0.21 ± 0.01 ^c^	0.73 ± 0.09 ^a^	0.3 ± 0.01 ^c^	0.25 ± 0.02 ^c^	0.53 ± 0.02 ^b^	0.59 ± 0.03 ^b^	pine, polish, wood
(*E*)-pent-2-enal-M	C1576870	1110.7	1.09998	0.68 ± 0.06 ^c^	0.45 ± 0.01 ^e^	2.05 ± 0.09 ^a^	0.61 ± 0.05 ^cd^	1.23 ± 0.02 ^b^	0.53 ± 0.03 ^de^	green, fruit, herb
(*E*)-pent-2-enal-D	C1576870	1110.4	1.37305	0.13 ± 0.01 ^c^	0.09 ± 0.01 ^c^	1.34 ± 0.16 ^a^	0.16 ± 0.01 ^bc^	0.32 ± 0.02 ^b^	0.32 ± 0.01 ^b^	green, fruit, herb
2-methylpropan-1-ol-M	C78831	1102.7	1.178	1.16 ± 0.07 ^c^	0.77 ± 0.06 ^d^	0.59 ± 0.04 ^e^	1.43 ± 0.02 ^b^	1.66 ± 0.06 ^a^	1.38 ± 0.02 ^b^	apple, bitter, wine
2-methylpropan-1-ol-D	C78831	1102.4	1.37305	0.59 ± 0.04 ^c^	0.28 ± 0.02 ^e^	0.37 ± 0.00 ^d^	1.25 ± 0.04 ^b^	0.66 ± 0.02 ^c^	2.1 ± 0.05 ^a^	apple, bitter, wine
hexanal-M	C66251	1097	1.26304	1.63 ± 0.11 ^c^	1.97 ± 0.07 ^b^	2.79 ± 0.02 ^a^	1.49 ± 0.03 ^c^	2.13 ± 0.05 ^b^	1.19 ± 0.03 ^d^	apple, fat, fresh, green
hexanal-D	C66251	1097	1.5876	0.67 ± 0.12 ^c^	0.86 ± 0.05 ^b^	2.56 ± 0.09 ^a^	0.56 ± 0.01 ^c^	0.93 ± 0.01 ^b^	0.25 ± 0.06 ^d^	apple, fat, fresh, green
butylcyclohexane	C1678939	1077.3	1.26148	0.37 ± 0.06 ^a^	0.05 ± 0.00 ^b^	0.07 ± 0.01 ^b^	0.46 ± 0.04 ^a^	0.09 ± 0.00 ^b^	0.09 ± 0.01 ^b^	-
ethyl 2-methylbutanoate	C7452791	1061.8	1.24588	0.37 ± 0.05 ^c^	0.07 ± 0.00 ^e^	0.23 ± 0.01 ^d^	0.61 ± 0.01 ^a^	0.09 ± 0.00 ^e^	0.5 ± 0.03 ^b^	fruit
propan-1-ol-M	C71238	1050.1	1.1148	0.5 ± 0.01 ^b^	0.94 ± 0.03 ^a^	0.53 ± 0.01 ^b^	0.52 ± 0.02 ^b^	0.99 ± 0.04 ^a^	0.53 ± 0.00 ^b^	alcohol, candy, pungent
propan-1-ol-D	C71238	1048.9	1.25758	0.37 ± 0.02 ^d^	0.39 ± 0.02 ^d^	0.33 ± 0.01 ^d^	0.77 ± 0.00 ^b^	0.6 ± 0.04 ^c^	1.5 ± 0.02 ^a^	alcohol, candy, pungent
ethyl butanoate-M	C105544	1048.9	1.21233	0.8 ± 0.02 ^b^	0.11 ± 0.01 ^e^	0.95 ± 0.04 ^a^	0.61 ± 0.00 ^c^	0.34 ± 0.01 ^d^	0.29 ± 0.01 ^d^	fruit, butter
ethyl butanoate-D	C105544	1047.3	1.5837	0.92 ± 0.12 ^b^	0.05 ± 0.01 ^c^	0.98 ± 0.09 ^b^	2.05 ± 0.04 ^a^	0.08 ± 0.01 ^c^	0.06 ± 0 ^c^	fruit, butter
ID_2	unidentified	1024.5	1.19672	2.29 ± 0.09 ^b^	1.59 ± 0.07 ^d^	1.67 ± 0.04 ^cd^	2.35 ± 0.01 ^a^	1.27 ± 0.03 ^e^	1.75 ± 0.06 ^c^	-
ethanol	C64175	943.7	1.13899	36.66 ± 0.2 ^a^	31.21 ± 1.06 ^c^	31.23 ± 0.25 ^c^	34.03 ± 0.30 ^b^	27.73 ± 0.03 ^d^	34.08 ± 0.08 ^b^	alcohol
ethyl 2-methylpropanoate	C97621	991.3	1.20609	1.18 ± 0.06 ^a^	0.32 ± 0.04 ^c^	0.74 ± 0.01 ^b^	0.79 ± 0.05 ^b^	1.28 ± 0.14 ^a^	0.13 ± 0.01 ^d^	fruit
ID_3	unidentified	965.4	1.47603	0.64 ± 0.05 ^b^	0.06 ± 0.00 ^c^	0.14 ± 0.00 ^c^	1.51 ± 0.10 ^a^	0.11 ± 0.00 ^c^	0.05 ± 0.01 ^c^	-
2-methylbutanal	C96173	921.8	1.4222	1.65 ± 0.04 ^a^	1.03 ± 0.04 ^c^	0.5 ± 0.02 ^e^	1.2 ± 0.04 ^b^	0.42 ± 0.07 ^e^	0.62 ± 0.03 ^d^	almond, nut, fermented
butan-2-one-M	C78933	909.5	1.06877	0.27 ± 0.01 ^bc^	0.17 ± 0.00 ^d^	0.31 ± 0.01 ^ab^	0.23 ± 0.02 ^c^	0.33 ± 0.01 ^a^	0.17 ± 0.04 ^d^	fragrant, fruit, pleasant
butan-2-one-D	C78933	908.8	1.26226	0.18 ± 0.00 ^c^	0.04 ± 0.00 ^e^	0.27 ± 0.02 ^a^	0.15 ± 0.01 ^d^	0.2 ± 0.00 ^b^	0.05 ± 0.01 ^e^	fragrant, fruit, pleasant
ethyl acetate-M	C141786	901.2	1.11246	0.98 ± 0.02 ^b^	1.11 ± 0.02 ^a^	1.02 ± 0.02 ^b^	0.84 ± 0.02 ^c^	1.18 ± 0.03 ^a^	0.86 ± 0.05 ^c^	aromatic, brandy, grape
ethyl acetate-D	C141786	893.5	1.35354	7.48 ± 0.03 ^c^	8.9 ± 0.14 ^b^	9.35 ± 0.14 ^bc^	7.2 ± 0.08 ^c^	9.73 ± 0.08 ^a^	7.63 ± 0.34 ^c^	aromatic, brandy, grape
ID_4	unidentified	860.8	1.07111	2.55 ± 0.07 ^a^	0.7 ± 0.05 ^c^	1.22 ± 0.06 ^b^	2.44 ± 0.07 ^a^	0.75 ± 0.01 ^c^	0.72 ± 0.05 ^c^	-
methyl acetate-M	C79209	846.7	1.03905	0.49 ± 0.02 ^de^	0.82 ± 0.02 ^b^	0.71 ± 0.01 ^c^	0.45 ± 0.02 ^e^	0.89 ± 0.02 ^a^	0.53 ± 0.01 ^d^	ester, green
methyl acetate-D	C79209	845.5	1.20826	0.93 ± 0.01 ^e^	1.48 ± 0.02 ^c^	2.22 ± 0.04 ^a^	1.13 ± 0.02 ^d^	1.64 ± 0.05 ^b^	1.6 ± 0.06 ^b^	ester, green
oct-1-ene	C111660	841.6	1.16972	0.59 ± 0.01 ^b^	0.56 ± 0.02 ^bc^	0.69 ± 0.02 ^a^	0.54 ± 0.02 ^cd^	0.66 ± 0.01 ^a^	0.5 ± 0.02 ^d^	-
propan-2-one	C67641	836	1.12921	1.96 ± 0.01 ^a^	1.24 ± 0.03 ^d^	1.45 ± 0.06 ^b^	1.54 ± 0.11 ^b^	1.46 ± 0.05 ^b^	1.17 ± 0.06 ^d^	pungent
butanal-M	C123728	832.3	1.10066	0.86 ± 0.01 ^b^	1.06 ± 0.06 ^a^	0.6 ± 0.01 ^e^	0.72 ± 0.02 ^cd^	0.75 ± 0.04 ^c^	0.63 ± 0.02 ^de^	
butanal-D	C123728	831.3	1.29777	0.92 ± 0.06 ^a^	0.93 ± 0.08 ^a^	0.27 ± 0.01 ^d^	0.72 ± 0.05 ^b^	0.33 ± 0.03 ^d^	0.56 ± 0.07 ^c^	banana, green, pungent
propanal-M	C123386	819.1	1.07717	0.87 ± 0.02 ^b^	0.9 ± 0.04 ^b^	1.16 ± 0.05 ^a^	0.81 ± 0.02 ^b^	1.16 ± 0.05 ^a^	0.46 ± 0.04 ^c^	
propanal-D	C123386	819.1	1.15534	0.74 ± 0.08 ^b^	0.52 ± 0.04 ^c^	0.68 ± 0.04 ^b^	0.95 ± 0.02 ^a^	0.93 ± 0.03 ^a^	0.22 ± 0.02 ^d^	floral, pungent
ID_5	unidentified	795.6	1.13182	0.93 ± 0.02 ^b^	0.18 ± 0.01 ^e^	0.58 ± 0.01 ^c^	1.05 ± 0.02 ^a^	0.12 ± 0.01 f	0.24 ± 0.01 ^d^	-
ID_6	unidentified	1428	1.09417	2.14 ± 0.12 ^bc^	2.55 ± 0.23 ^ab^	2.76 ± 0.20 ^a^	2.1 ± 0.08 ^bc^	2.47 ± 0.26 ^ab^	1.75 ± 0.09 ^d^	-
octanal	C124130	1298.7	1.41383	0.22 ± 0.03 ^c^	0.28 ± 0.02 ^b^	0.34 ± 0.02 ^a^	0.16 ± 0.01 ^d^	0.31 ± 0.01 ^ab^	0.14 ± 0.01 ^d^	citrus, fat, green
*β*-Ocimene	C13877913	1248.5	1.23231	0.22 ± 0.01 ^c^	1.13 ± 0.25 ^b^	0.38 ± 0.04 ^c^	0.25 ± 0.01 ^c^	1.67 ± 0.10 ^a^	1.21 ± 0.06 ^b^	floral
(*E*)-hex-2-enal	C6728263	1226.7	1.19301	0.18 ± 0.02 ^c^	0.34 ± 0.03 ^b^	0.52 ± 0.02 ^a^	0.13 ± 0.01 ^cd^	0.34 ± 0.04 ^b^	0.11 ± 0.00 ^d^	green, fruit, grass
pentan-1-ol	C71410	1267.9	1.25106	0.12 ± 0.02 ^b^	0.1 ± 0.01 ^bc^	0.13 ± 0.01 ^b^	0.27 ± 0.02 ^a^	0.11 ± 0.01 ^b^	0.07 ± 0.01 ^c^	fruit, green
3-methylpentan-1-ol	C589355	1353.5	1.32182	0.12 ± 0.01 ^b^	0.1 ± 0.03 ^b^	0.19 ± 0.03 ^a^	0.21 ± 0.02 ^a^	0.1 ± 0.01 ^b^	0.06 ± 0.01 ^b^	fruit
*α*-terpinolene	C586629	1286.7	1.23608	0.12 ± 0.02 ^c^	0.32 ± 0.05 ^b^	0.18 ± 0.01 ^c^	0.13 ± 0.00 ^c^	0.49 ± 0.02 ^a^	0.47 ± 0.03 ^a^	pine
styrene-M	C100425	1266	1.06685	0.21 ± 0.01 ^d^	0.3 ± 0.02 ^c^	0.4 ± 0.01 ^b^	0.28 ± 0.03 ^c^	0.48 ± 0.02 ^a^	0.28 ± 0.02 ^c^	-
styrene-D	C100425	1266.4	1.47187	0.09 ± 0.01 ^d^	0.13 ± 0.02 ^c^	0.17 ± 0.01 ^b^	0.11 ± 0.00 ^cd^	0.24 ± 0.02 ^a^	0.09 ± 0.01 ^d^	-
*α*-terpinene	C99865	1183.4	1.23244	0.11 ± 0.00 ^c^	0.66 ± 0.16 ^b^	0.25 ± 0.02 ^c^	0.14 ± 0.00 ^c^	1.09 ± 0.09 ^a^	0.75 ± 0.03 ^b^	lemon
butyl acetate	C123864	1084.5	1.25013	0.24 ± 0.02 ^b^	0.12 ± 0.03 ^c^	0.31 ± 0.01 ^b^	0.3 ± 0.02 ^b^	0.67 ± 0.01 ^a^	0.27 ± 0.08 ^b^	apple, banana
propyl butanoate	C105668	1143.5	1.27774	0.08 ± 0.01 ^b^	0.05 ± 0.01 ^c^	0.08 ± 0.01 ^b^	0.24 ± 0.00 ^a^	0.05 ± 0.00 ^c^	0.05 ± 0.00 ^c^	apricot, fruit, pineapple
*α*-pinene	C80568	1031.7	1.23215	0.41 ± 0.01 ^c^	1.19 ± 0.18 ^b^	0.52 ± 0.04 ^c^	0.54 ± 0.03 ^c^	1.18 ± 0.06 ^b^	1.47 ± 0.11 ^a^	cedarwood, pine
ID_7	unidentified	1023.9	1.31129	0.21 ± 0.01 ^c^	0.11 ± 0.01 ^c^	0.21 ± 0.03 ^c^	0.43 ± 0.01 ^b^	0.77 ± 0.02 ^a^	0.77 ± 0.13 ^a^	-
2-methylbutan-1-ol-D	C137326	1215.8	1.49253	0.22 ± 0.02 ^c^	0.24 ± 0.03 ^bc^	0.23 ± 0.01 ^bc^	0.34 ± 0.00 ^b^	0.34 ± 0.03 ^b^	2.32 ± 0.09 ^a^	green, wine
2-methylbutan-1-ol-M	C137326	1214.7	1.23582	0.48 ± 0.05 ^e^	0.8 ± 0.10 ^c^	0.56 ± 0.04 ^de^	0.66 ± 0.03 ^cd^	1.42 ± 0.08 ^b^	2.05 ± 0.05 ^a^	green, wine

Note: ID_1-ID_7, represent unknown volatile compounds; RI, retention index; Dt, drift time; M, monomer; D, dimer; T, trimer. Each value is expressed as the mean ± SD of triplicate determinations. Significant differences among samples from different origins were determined via ANOVA. Different letters within a row indicate a significant difference (*p* < 0.05). * Odor descriptions were obtained from https://www.femaflavor.org/flavor-library accessed on 20 August 2025. “-” indicates that the no odor description was available for the compound. In addition, some compounds are widely recognized by their common names. For clarity, these common names have been retained, and their corresponding IUPAC names are provided as follows: (+)-limonene: 1-methyl-4-prop-1-en-2-ylcyclohexene. (*Z*)-Ocimene: (3*Z*)-3,7-dimethylocta-1,3,6-triene. delta-3-carene: 3,7,7-trimethylbicyclo[4.1.0]hept-3-ene. *β*-Pinene: 6,6-dimethyl-2-methylidenebicyclo[3.1.1]heptane. *β*-Ocimene: 3,7-dimethylocta-1,3,6-triene. *α*-Terpinolene: 1-methyl-4-propan-2-ylidenecyclohexene. *α*-Terpinene: 1-methyl-4-propan-2-ylcyclohexa-1,3-diene. α-Pinene: 2,6,6-trimethylbicyclo[3.1.1]hept-2-ene.

**Table 3:** Relative contents of volatile compounds in pomegranates of different origins via HS-SPME-GC-MS.

No.	Compounds	CAS	RI	Relative Content (%)					
				MZ	HY	LT	TNS	HL	HZZ
**Alcohols**									
1	terpinen-4-ol	562-74-3	1617	2.55 ± 0.08 ^d^	0.54 ± 0.05 ^f^	9.66 ± 0.05 b	1.86 ± 0.4 ^e^	11.75 ± 0.25 a	7.12 ± 0.11 ^c^
2	menthol	15356-70-4	1544	ND	ND	6.05 ± 0.04 ^a^	ND	ND	ND
3	4-methylpentan-1-ol	626-89-1	1316	5.87 ± 0.4 ^a^	ND	ND	5.38 ± 0.03 ^b^	ND	ND
4	linalool	78-70-6	1556	ND	ND	ND	ND	3.7 ± 0.11 a	0.83 ± 0.02 ^b^
5	2-phenylethanol	60-12-8	1935	ND	5.43 ± 0.05 ^a^	1.07 ± 0.12 c	0.81 ± 0.02 ^d^	1.51 ± 0.36 ^b^	1.44 ± 0.26 ^b^
6	*β*-bisabolol	15352-77-9	2162	ND	ND	ND	ND	2.94 ± 0.6 a	ND
7	(*E*)-dec-2-en-1-ol	18409-18-2	1819	ND	ND	ND	ND	0.57 ± 0.32 a	ND
8	2-methylbut-3-en-2-ol	115-18-4	1048	ND	ND	ND	ND	0.91 ± 0.03 a	0.92 ± 0.02 ^a^
9	nonan-1-ol	143-08-8	1664	ND	ND	ND	4.62 ± 0.02 a	ND	ND
10	3-methylbut-2-en-1-ol	556-82-1	1327	ND	ND	ND	ND	0.93 ± 0.03 ^a^	0.51 ± 0.04 ^b^
11	heptan-2-ol	543-49-7	1328	ND	0.41 ± 0.01 ^a^	ND	ND	ND	ND
12	nonan-2-ol	628-99-9	1521	ND	6.28 ± 0.07 ^a^	ND	ND	ND	ND
**Aldehydes**									
13	3-methylbutanal	590-86-3	918	ND	ND	1.69 ± 0.08 ^a^	ND	ND	ND
14	hexanal	66-25-1	1093	2.38 ± 0.07 ^c^	ND	6.30 ± 0.28 ^a^	2.91 ± 0.5 b	2.95 ± 0.52 b	ND
15	nonanal	124-19-6	1389	1.1 ± 0.19 b	ND	7.19 ± 0.09 ^a^	ND	0.57 ± 0.11 c	ND
16	furan-2-carbaldehyde	98-01-1	1482	ND	ND	5.25 ± 0.22 ^a^	ND	4.45 ± 0.08 ^b^	ND
17	5-methylfuran-2-carbaldehyde	620-02-0	1570	ND	ND	ND	ND	0.27 ± 0.01 a	ND
**Esters**									
18	ethyl acetate	141-78-6	894	14.01 ± 0.21 ^b^	28.8 ± 1.12 ^a^	12.01 ± 0.13 c	6.63 ± 0.15 d	3.62 ± 0.36 ^e^	6.74 ± 0.9 d
19	ethyl butanoate	105-54-4	1030	1.85 ± 0.08 a	ND	1.07 ± 0.09 ^b^	1.74 ± 0.03 a	ND	ND
20	ethyl (*E)*-but-2-enoate	623-70-1	1154	1.81 ± 0.07 ^a^	ND	0.82 ± 0.07 b	ND	ND	ND
21	ethyl hexanoate	123-66-0	1241	3.21 ± 0.15 ^b^	0.93 ± 0.04 d	1.20 ± 0.06 c	3.63 ± 0.6 a	ND	ND
22	dec-9-enyl acetate	50816-18-7	1722	ND	ND	3.15 ± 0.06 a	ND	0.98 ± 0.08 b	ND
23	methyl 2-hydroxybenzoate	119-36-8	1751	1.00 ± 0.01 ^b^	ND	2.39 ± 0.06 a	ND	ND	ND
24	geranyl hexanoate	10032-02-7	1726	ND	ND	ND	ND	0.42 ± 0.04 ^a^	ND
25	3-methylbutyl acetate	123-92-2	1137	ND	13.94 ± 1.7 ^a^	ND	ND	ND	ND
26	ethyl octanoate	106-32-1	1430	ND	6.99 ± 0.11 ^a^	ND	ND	ND	ND
27	nonan-2-yl acetate	14936-66-4	1425	ND	0.48 ± 0.01 ^a^	ND	ND	ND	ND
28	ethyl decanoate	110-38-3	1645	ND	23.82 ± 0.52 ^a^	ND	ND	ND	0.83 ± 0.06 ^b^
29	ethyl dodecanoate	106-33-2	1835	ND	4.71 ± 0.06 ^a^	ND	ND	ND	ND
**Ketones**									
30	acetoin	513-86-0	1277	28.15 ± 0.61 ^a^	2.10 ± 0.19 ^c^	1.63 ± 0.03 ^d^	26.49 ± 1.25 ^b^	ND	1.01 ± 0.01 ^e^
31	hexan-2-one	591-78-6	1078	1.37 ± 0.06 ^a^	ND	ND	ND	ND	ND
32	6-methylhept-5-en-2-one	110-93-0	1348	1.83 ± 0.07 ^a^	ND	ND	ND	0.56 ± 0.08 b	ND
33	propan-2-one	67-64-1	821	ND	ND	ND	ND	0.44 ± 0.01 b	0.96 ± 0.01 a
34	(5*Z*)-6,10-dimethylundeca-5,9-dien-2-one	3879-26-3	1813	ND	ND	1.13 ± 0.01 a	ND	ND	ND
**Alkenes**									
35	(*Z*,*E*)-*α*-Farnesene	26560-14-5	1737	1.15 ± 0.01 ^d^	0.54 ± 0.02 ^e^	1.26 ± 0.01 d	2.06 ± 0.06 c	2.39 ± 0.06 b	5.96 ± 0.07 a
36	D-Limonene	5989-27-5	1205	2.82 ± 0.03 c	1.45 ± 0.05 ^e^	4.03 ± 0.03 b	2.69 ± 0.12 d	6.39 ± 0.2 a	6.35 ± 0.04 a
37	(*E*)-dec-4-ene	19398-89-1	982	ND	ND	0.97 ± 0.01 a	ND	ND	ND
38	*α*-Curcumene	644-30-4	1784	ND	ND	ND	1.68 ± 0.05 b	1.47 ± 0.08 c	2.86 ± 0.07 a
39	*β*-Curcumene	28976-67-2	1756	9.59 ± 0.07 ^d^	3.25 ± 0.21 ^e^	13.51 ± 0.06 ^c^	17.91 ± 0.4 b	17.52 ± 0.07 b	41.52 ± 0.03 a
40	(*E*)-4-methylhept-3-ene	4485-16-9	885	ND	ND	ND	ND	0.18 ± 0.01 a	ND
41	*β*-Bisabolene	495-61-4	1743	1.71 ± 0.05 ^c^	ND	ND	5.26 ± 0.12 a	ND	2.90 ± 0.1 ^b^
42	(*E*)-tetradec-7-ene	10374-74-0	1365	ND	ND	ND	ND	1.35 ± 0.15 a	ND
43	nonadec-1-ene	18435-45-5	1922	ND	ND	ND	ND	0.31 ± 0.05 b	0.64 ± 0.02 a
44	*γ*-Muurolene	30021-74-0	1704	ND	ND	ND	ND	0.21 ± 0.01 a	ND
45	*γ*-Terpinene	99-85-4	1255	ND	0.38 ± 0.01 ^c^	ND	ND	3.74 ± 0.12 ^a^	0.70 ± 0.05 b
46	(*E*)-*β*-Famesene	18794-84-8	1673	ND	ND	ND	2.16 ± 0.02 ^a^	1.78 ± 0.11 ^b^	0.90 ± 0.01 c
47	(+)-4-Carene	29050-33-7	1128	ND	ND	ND	ND	2.31 ± 0.04 ^a^	0.47 ± 0.02 b
48	styrene	100-42-5	1225	8.52 ± 0.06 c	0.87 ± 0.06 ^f^	7.90 ± 0.64 ^d^	10.15 ± 0.13 b	15.36 ± 0.23 a	1.13 ± 0.02 e
**Acids and Ethers**									
49	acetic acid	64-19-7	1471	ND	ND	ND	ND	0.89 ± 0.06 b	12.84 ± 0.19 a
50	nonanoic acid	112-05-0	2184	3.08 ± 0.05 ^a^	ND	2.08 ± 0.04 b	ND	2.12 ± 0.15 b	ND
51	2-methylbutanoic acid	116-53-0	1688	ND	ND	1.67 ± 0.15 a	ND	ND	ND
52	octanoic acid	124-07-2	2035	ND	0.81 ± 0.07 ^a^	ND	ND	ND	ND
53	eucalyptol	470-82-6	1216	2.94 ± 0.24 ^b^	ND	1.47 ± 0.12 c	3.21 ± 0.02 a	0.34 ± 0.05 d	ND
**Phenols**									
54	phenol	108-95-2	2037	1.51 ± 0.3 ^b^	ND	2.63 ± 0.06 a	0.82 ± 0.02 d	1.18 ± 0.13 c	0.58 ± 0.05 ^e^
55	2-methoxyphenol	90-05-1	1859	1.98 ± 0.06 ^a^	ND	ND	ND	ND	ND
56	4-ethenyl-2-methoxyphenol	7786-61-0	2180	ND	ND	0.92 ± 0.07 ^a^	ND	0.67 ± 0.06 ^b^	ND
**Hydrocarbons**									
57	cyclododecane	294-62-2	1519	ND	ND	2.95 ± 0.05 a	ND	ND	ND
58	*p*-Cymene	99-87-6	1280	1.35 ± 0.01 ^c^	ND	ND	ND	4.66 ± 0.04 a	2.78 ± 0.12 b

Note: RT, Retention time; ND, not detected. Each value is expressed as the mean ± SD of triplicate determinations. Significant differences among samples from different origins were determined via ANOVA. Different letters within a row indicate a significant difference (*p* < 0.05). Some compounds in the table are presented by their common names; the corresponding IUPAC names are provided in Table S1.

**Table 4:** ROAVs and odor descriptions of main volatile compounds in pomegranates analyzed via HS-SPME-GC-IMS and HS-SPME-GC-MS.

	Compounds	Threshold ^#^ (mg/kg)	ROAV						Odor Description *
			MZ	HY	LT	TNS	HL	HZZ	
HS-GC-IMS	nonanal	0.0011	10.37	43.13	28.14	4.79	11.88	4.47	floral, green, lemon
	3-hydroxybutan-2-one	0.0140	1.52	3.09	3.03	1.53	1.34	1.30	butter, creamy, green pepper
	heptanal	0.0028	1.09	4.42	2.35	0.48	1.23	0.36	citrus, fat, green, nut
	*α*-phellandrene	0.0400	0.14	1.65	0.25	0.11	0.55	0.43	citrus, fresh, mint, pepper, wood
	2-butylfuran	0.0050	1.50	5.64	2.25	0.74	1.86	0.73	wet hay
	*β*-pinene	0.1400	0.14	1.68	0.34	0.11	0.35	0.28	pine, polish, wood
	hexanal	0.073	0.38	1.86	1.05	0.21	0.5	0.21	apple, fat, fresh, green
	ethyl 2-methylbutanoate	0.000063	100.00	76.39	100.00	100.00	24.55	100.00	apple, ester, green apple, kiwi, strawberry
	ethyl butanoate-M	0.0030	4.54	2.52	8.67	2.1	1.95	1.22	apple, butter, cheese, pineapple, strawberry
	ethyl butanoate-D	0.0030	5.22	1.15	8.95	7.06	0.46	0.25	apple, butter, cheese, pineapple, strawberry
	ethyl 2-methylpropionate	0.0002	91.33	100	92.13	37.09	100	7.45	fruit, sweet
	2-methylbutanal	0.0044	6.39	16.09	3.11	2.82	1.64	1.78	almond, nut, fermented
	oct-1-ene	0.0005	20.09	77.00	37.80	11.15	22.69	12.60	-
	butanal-M	0.0020	7.32	36.44	8.22	3.72	6.45	3.97	banana, green, pungent
	butanal-D	0.0020	7.83	31.97	3.7	3.72	2.84	3.53	banana, green, pungent
	propanal-M	0.0151	0.98	4.10	2.10	0.55	1.32	0.38	floral, pungent
	propanal-D	0.0151	0.83	2.37	1.23	0.65	1.06	0.18	floral, pungent
	octanal	0.0069	0.54	2.79	1.35	0.24	0.77	0.26	citrus, fat, green, pungent
	*β*-ocimene	0.0340	0.11	2.28	0.31	0.08	0.84	0.45	floral
	*α*-pinene	0.0410	0.17	2.00	0.35	0.14	0.49	0.45	cedarwood, pine
HS-SPME-GC-MS	nonan-1-ol	0.087	ND	ND	ND	2.81	ND	ND	floral, green, fat
	3-methylbutanal	0.0012	ND	ND	58.63	ND	ND	ND	-
	hexanal	0.073	1.16	ND	3.59	2.11	5.58	ND	apple, fat, fresh, green
	nonanal	0.008	4.91	ND	37.42	ND	9.84	ND	floral, green, lemon
	ethyl acetate	0.005	100	2.42	100	70.08	100	16.24	aromatic, brandy, grape
	ethyl butanoate	0.003	22.01	ND	14.85	30.65	ND	ND	apple, butter, cheese, pineapple, strawberry
	ethyl hexanoate	0.005	22.91	0.08	9.99	38.37	ND	ND	fruit
	methyl 2-hydroxybenzoate	0.04	0.89	ND	2.49	ND	ND	ND	
	3-methylbutyl acetate	0.002	ND	2.93	ND	ND	ND	ND	apple, banana, pear
	ethyl decanoate	0.0001	ND	100	ND	ND	ND	100	brandy, grape, pear
	acetoin	0.014	71.76	0.06	4.85	100	ND	0.87	butter, creamy, green pepper
	(*Z*,*E*)-*α*-farnesene	0.087	0.47	ND	0.60	1.25	3.79	5.08	floral, wood
	D-limonene	0.034	2.96	0.02	4.93	4.18	25.96	2.25	citrus, mint
	styrene	0.065	4.68	0.01	5.06	8.25	32.64	0.21	-
	eucalyptol	0.023	4.56	ND	2.42	7.38	2.04	ND	
	2-methoxyphenol	0.0016	44.16	ND	ND	ND	ND	ND	burnt, phenol, wood
	p-cymene	0.01	0.48	ND	ND	ND	6.44	0.33	citrus, fresh

Note: ^#^ Odor thresholds for volatile compounds were obtained from http://www.odour.org.uk accessed on 20 August 2025 and the Compilations of Odor Threshold Values in Air, Water, and Other Media. * Odor descriptions were obtained from https://www.femaflavor.org/flavor-library accessed on 21 August 2025. The average relative content was used for calculation. ND: Not detected. “-” indicates that the no odor description was available for the compound.

## Data Availability

The original contributions presented in the study are included in the article/Supplementary Materials; further inquiries can be directed to the corresponding authors.

## Supplementary Materials

The following supporting information can be downloaded at https://www.mdpi.com/article/10.3390/foods14203546/s1. Figure S1: Analysis of pomegranate samples from different origins by electronic technology. (A) LDA score plot and (B) VIP values plot by the electronic nose. (C) LDA score plot and (D) VIP values plot by the electronic tongue. Note: the red bar (VIP > 1) indicates significant differences in aroma and taste among pomegranate origins, Figure S2: Comparison of VOCs detected by HS-GC-IMS and HS-SPME-GC-MS. (A) Classification and number of VOCs identified by each technique. (B) Venn diagram showing the overlap and unique VOCs detected by the two analytical methods, Table S1: Common names and corresponding IUPAC names of volatile compounds identified by HS-SPME-GC-MS in this study.
