# Supplementary material for: Identification of Key Aroma Substances in Pomegranate from Different Geographical Origins via Integrated Volatile Profiling and Multivariate Statistical Analysis
Source: Foods. 2025 Oct 17;14(20):3546. doi: 10.3390/foods14203546 (PMC12563161; doi:10.3390/foods14203546)
Supplement: Supplementary file 1 [file foods-14-03546-s001.zip › foods-3901466-supplementary.pdf]

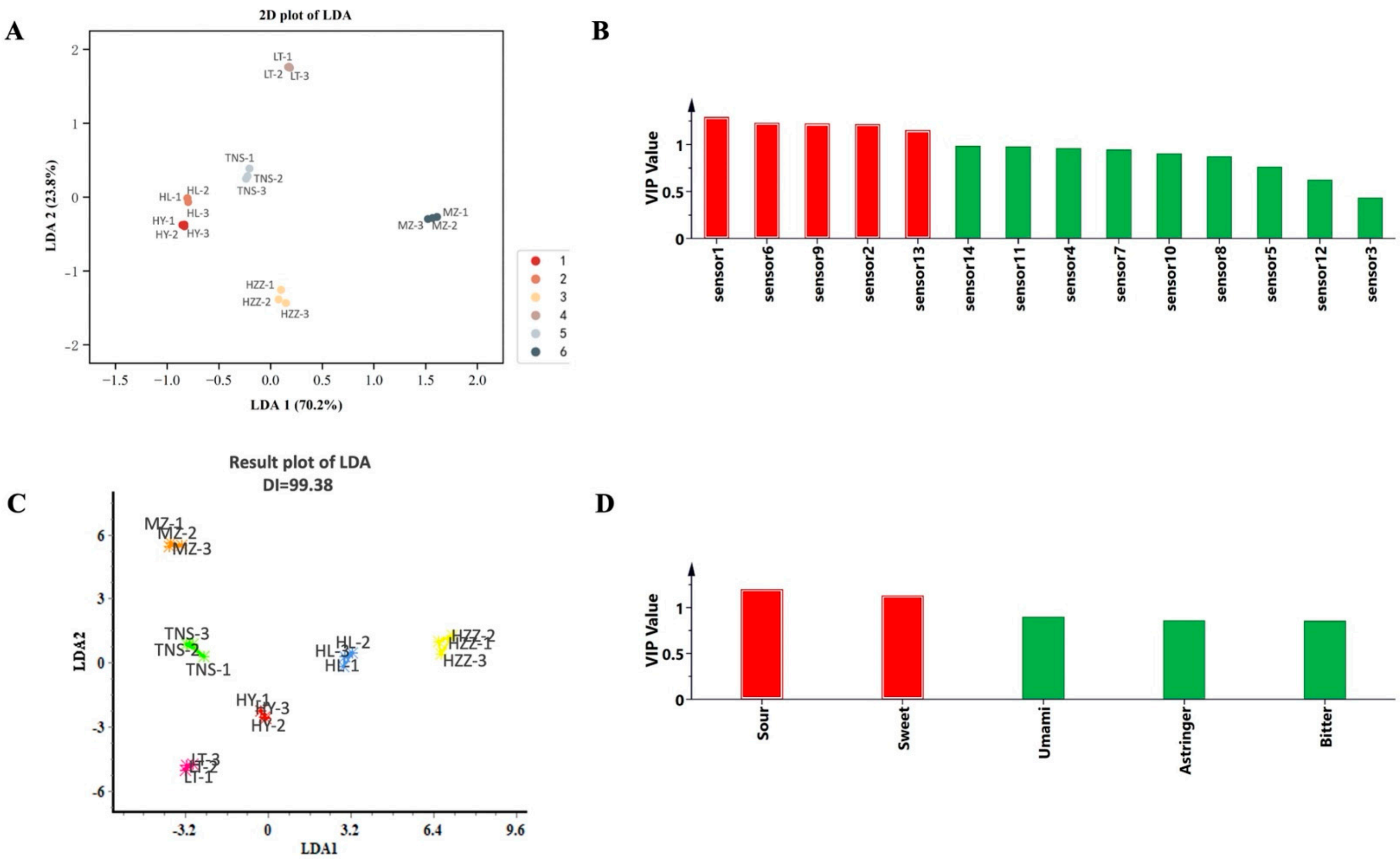

Figure S1. Analysis of pomegranate samples from different origins by electronic technology. (A) LDA score plot and (B) VIP values plot by the electronic nose. (C) LDA score plot and (D) VIP values plot by the electronic tongue. Red bar (VIP>1) indicates that the taste has important differences among pomegranate origins.

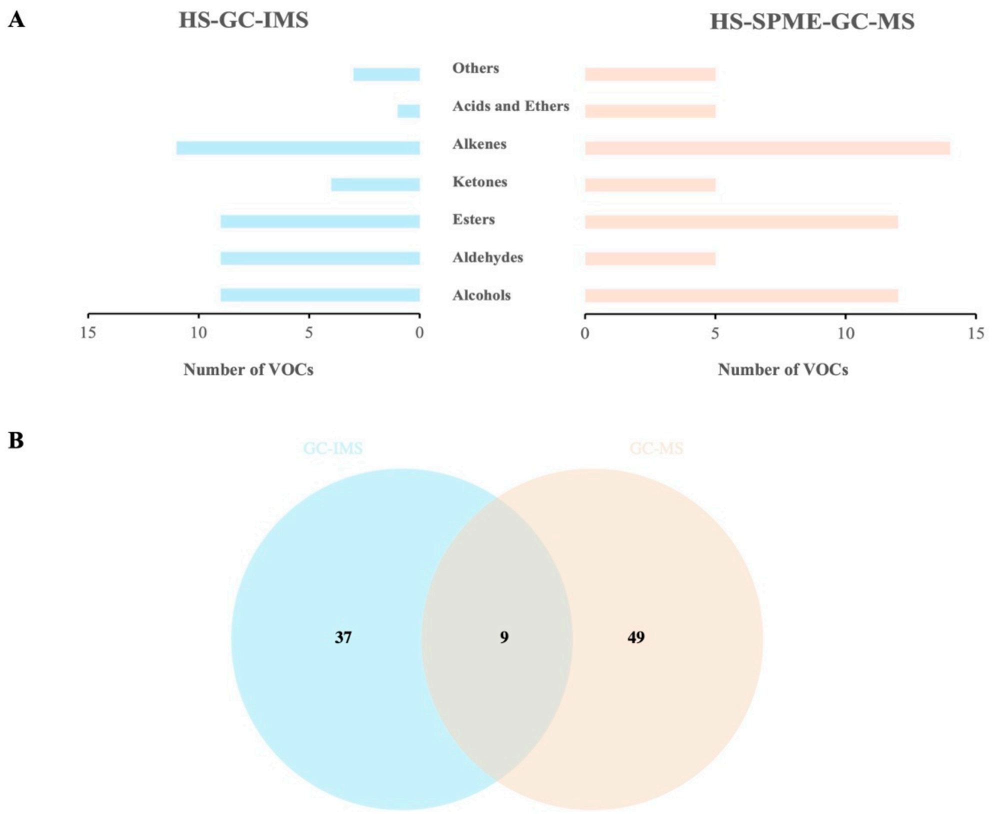

Figure S2. Comparison of VOCs detected by HS-GC-IMS and HS-SPME-GC-MS. (A) Classification and number of VOCs identified by each technique. (B) Venn diagram showing the overlap and unique VOCs detected by the two analytical methods.

Table S1. Common names and corresponding IUPAC names of volatile compounds identified by HS-SPME-GC-MS in this study.

No.	Common names	IUPAC names
1	terpinen-4-ol	4-methyl-1-propan-2-ylcyclohex-3-en-1-ol
2	menthol	5-methyl-2-propan-2-ylcyclohexan-1-ol
3	linalool	3,7-dimethylocta-1,6-dien-3-ol
4	*β*-bisabolol	(1*S*)-4-methyl-1-[(2*S*)-6-methylhept-5-en-2-yl]cyclohex-3-en-1-ol
5	geranyl hexanoate	[(2*E*)-3,7-dimethylocta-2,6-dienyl] hexanoate
6	acetoin	3-hydroxybutan-2-one
7	(*Z*,*E*)-*α*-Farnesene	(3*Z*,6*E*)-3,7,11-trimethyldodeca-1,3,6,10-tetraene
8	D-Limonene	(4*R*)-1-methyl-4-prop-1-en-2-ylcyclohexene
9	*α*-Curcumene	1-methyl-4-(6-methylhept-5-en-2-yl)benzene
10	*β*-Curcumene	1-methyl-4-[(2*R*)-6-methylhept-5-en-2-yl]cyclohexa-1,4-diene
11	*β*-Bisabolene	(4*S*)-1-methyl-4-(6-methylhepta-1,5-dien-2-yl)cyclohexene

12	*γ*-Muurolene	(1*S*,4*aS*,8*aR*)-7-methyl-4-methylidene-1-propan-2-yl-2,3,4*a*,5,6,8*a*-hexahydro-1*H*-naphthalene
13	*γ*-Terpinene	1-methyl-4-propan-2-ylcyclohexa-1,4-diene
14	(*E*)-*β*-Famesene	(6*E*)-7,11-dimethyl-3-methylidenedodeca-1,6,10-triene
15	(+)-4-Carene	4,7,7-trimethylbicyclo[4.1.0]hept-2-ene
16	eucalyptol	1,3,3-trimethyl-2-oxabicyclo[2.2.2]octane
17	*p*-Cymene	1-methyl-4-propan-2-ylbenzene
